# True *Schistosoma mansoni* eggs-cretome revealed by laser microdissection of infected mouse liver and intestine

**DOI:** 10.3389/fcimb.2026.1807773

**Published:** 2026-05-15

**Authors:** Lucie Jedličková, Lukáš Konečný, Jan Dvořák, Petr Horák, Tomáš Macháček

**Affiliations:** 1 Department of Parasitology, Faculty of Science, Charles University, Prague, Czechia; 2 Department of Ecology, Center of Infectious Animal Diseases, Faculty of Environmental Sciences, Czech University of Life Sciences, Prague, Czechia; 3 Institute of Organic Chemistry and Biochemistry, Czech Academy of Sciences, Prague, Czechia

**Keywords:** eggs, excretory and secretory products, granuloma, *in situ* proteomics, intestine, laser capture microdissection, liver, *Schistosoma mansoni*

## Abstract

**Introduction:**

*Schistosoma mansoni* eggs are the central drivers of schistosomiasis pathology and transmission, yet the proteins they release within host tissues remain poorly defined due to limitations of conventional *in vitro* approaches. Here, we provide the first *in situ* characterization of egg-derived proteins present in host tissues.

**Methods:**

Using laser microdissection, we selectively isolated periovular granuloma microenvironments from the livers and small intestines of infected mice during the acute and chronic phases of infection, followed by high-resolution mass spectrometry.

**Results:**

Across all samples, we identified a conserved core set of 149 parasite proteins, representing a true *in vivo* egg excretory/secretory repertoire. Egg-associated protein profiles varied markedly by organ and infection phase. In the liver, acute infection was characterized by a richer and more abundant repertoire of immunomodulators (including omega-1 and IPSE), proteases, protease inhibitors, antioxidant proteins, and micro-exon gene and venom allergen-like proteins, whereas chronic liver granulomas showed a reduced and altered profile. In contrast, intestinal granulomas exhibited fewer qualitative changes between phases and a more uniform protein composition over time, despite an overall decline in protein detectability. These patterns likely reflect fundamental differences in egg fate and tissue context, as hepatic eggs remain permanently trapped whereas intestinal eggs transit out of the host.

**Conclusion:**

This study provides the first *in situ* snapshot of schistosome egg-derived proteins within host tissues, revises assumptions about intestinal granuloma chronicity, and defines core and context-dependent egg proteins that are likely to shape granuloma biology, host modulation, and parasite transmission.

## 1 Introduction

Members of the genus *Schistosoma* are digenean parasitic flatworms belonging to the phylum Platyhelminthes, class Trematoda, and family Schistosomatidae. Schistosomes are exceptional among platyhelminths due to their gonochorism, distinct sexual dimorphism, and intravascular localization of adult worms in the definitive host. In humans, these parasites cause schistosomiasis – a serious yet neglected tropical disease reported in 78 countries, for which more than 250 million people required preventive treatment in 2021 (World Health Organisation, 2023).

The pathology of *Schistosoma mansoni* infection is driven mainly by the host’s immune response to parasite eggs lodged primarily in intestinal or hepatic tissues, where they trigger the formation of inflammatory granulomas (Costain et al., 2022). While intestinal granulomas facilitate the translocation of eggs through the intestinal wall into the lumen and ultimately to the external environment, hepatic granulomas formed around trapped eggs contribute significantly to severe pathology (Costain et al., 2018; Schwartz and Fallon, 2018). The interactions between *S. mansoni* eggs and host tissues are believed to be mainly facilitated by eggs’ excretory-secretory products (ESPs), which play pivotal roles in modulating the host’s immune system and shaping granuloma development (Ashton et al., 2001; Hagen et al., 2014; Ittiprasert et al., 2019). An accurate description of the *S. mansoni* egg excretome/secretome is therefore essential for understanding the intricate processes underlying successful egg release and disease progression.

The composition of helminth ESPs, including those of schistosomes, is traditionally characterized by *in vitro* cultivation of selected parasite stages, collection of released proteins, and subsequent proteomic analysis (Harnett, 2014; Leontovyč et al., 2020). However, this well-established experimental approach is prone to several sources of bias, including sample contamination by somatic proteins from damaged and dying/dead parasites due to *in vitro* conditions, artificial culture conditions that may alter the parasites’ physiology, and the absence of host tissue context during prolonged cultivation. Such limitations consequently hinder accurate identification of parasite-derived molecules that are genuinely excreted/secreted and biologically relevant at the parasite-host interface *in situ*. Due to these shortcomings, *S. mansoni* egg excretome/secretome remains poorly understood, as available proteomic studies vary considerably, both qualitatively and quantitatively (Cass et al., 2007; Mathieson and Wilson, 2010; Carson et al., 2020). Moreover, studies on schistosome eggs typically rely on eggs isolated from the host liver, as these are easier to obtain and less prone to bacterial contamination than intestinal eggs. Yet, recent studies have demonstrated that *S. mansoni* eggs isolated from the liver and intestine, as well as miracidia derived from these eggs, differ markedly in their gene expression profiles and behavior (Peterková et al., 2024; Willett et al., 2025), suggesting that distinct local host environments influence parasite gene regulation and, potentially, protein excretion/secretion. Whether these transcriptional differences are reflected at the proteomic level, specifically in the spectrum of molecules excreted/secreted within host tissues, remains unknown.

To address these unknowns, we employed a combination of laser capture microdissection and shotgun proteomics. This approach has been repeatedly demonstrated to be immensely useful for the precise isolation of particular host or parasite tissues and the characterization of protein/transcript composition while minimizing contamination from non-target material (Gobert et al., 2009; Nawaratna et al., 2011, 2014; Chuah et al., 2013; Macháček et al., 2022; Vondráček et al., 2022). Here, we applied this approach to the mouse liver and intestinal tissues, specifically to the periovular granuloma microenvironments, to identify *S. mansoni* proteins. This novel application enabled us to accurately characterize the true *in situ* repertoire of excreted/secreted parasite proteins present in egg-surrounding tissues, both in the chronic and acute phases of infection, while preserving the natural tissue context.

## 2 Materials and methods

### 2.1 Parasite material

The life cycle of *S. mansoni* (Puerto Rican strain) is routinely maintained in the Laboratory of Helminthology, Faculty of Science, Charles University (Prague, Czechia), using freshwater snails *Biomphalaria glabrata* and female ICR (CD-1) outbred mice (Envigo, Netherlands) as intermediate and definitive hosts, respectively. For the experiments, C57BL/6JOlaHsd inbred mice (7-week-old females; Envigo, Netherlands) were infected by exposure to 150 cercariae (acute phase) or 100 cercariae (chronic phase) per mouse in a water bath for one hour. Infected animals (n = 5 per time point) were sacrificed by anesthesia overdose at 7 weeks (acute phase) or 12 weeks (chronic phase) post-infection to collect infected tissues (liver and intestine) for further experiments.

### 2.2 Sample preparation for laser microdissection

**Figure 1:**
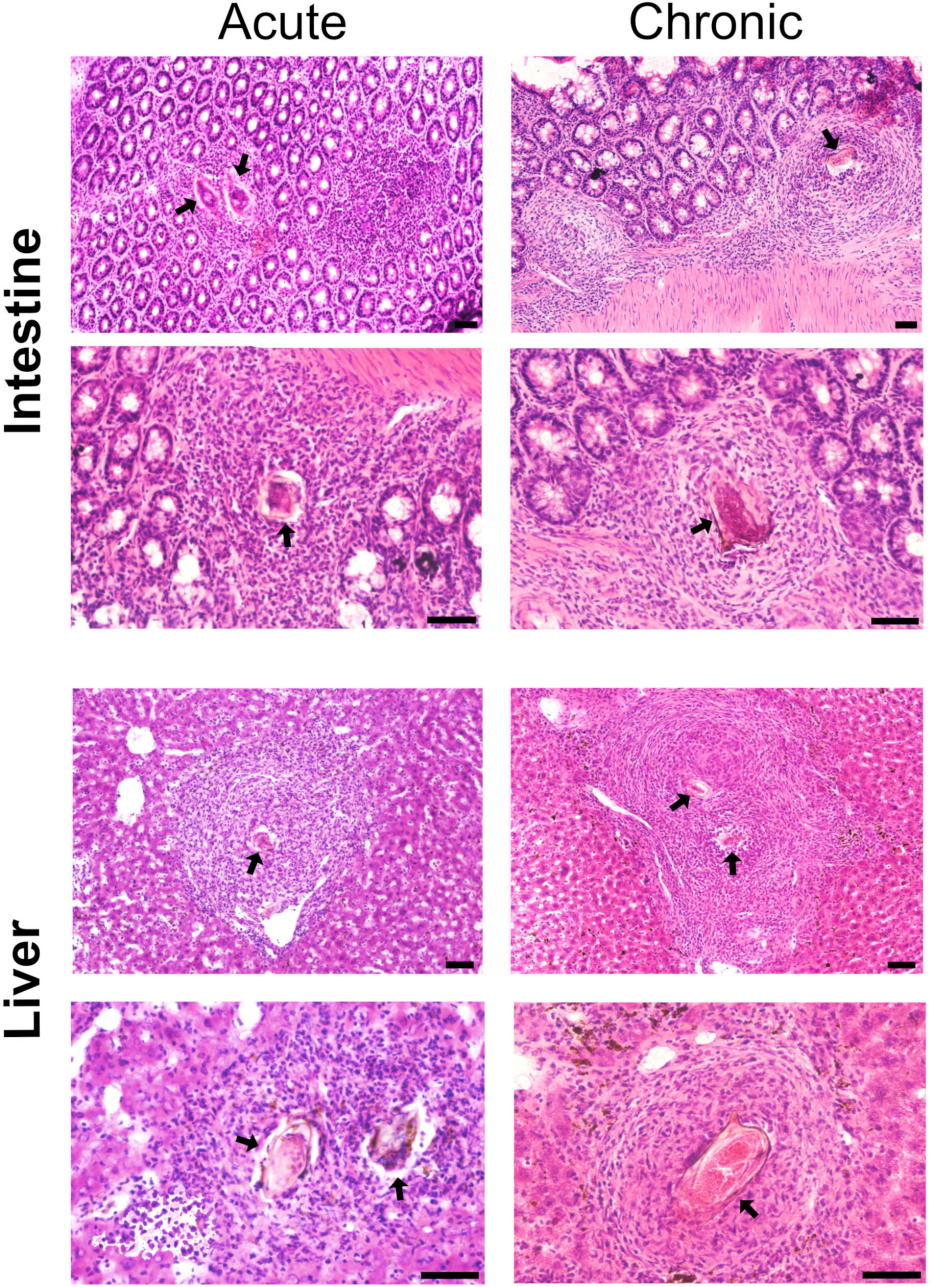
Histopathology of intestinal and hepatic granulomas in *Schistosoma mansoni* infected mice during acute and chronic infection. Representative cryosections (20 µm, stained by hematoxylin and eosin) of small intestine and liver tissues collected during the acute (left) and chronic (right) phases of *S. mansoni* infection. Granuloma structure and associated pathological changes are visible, with schistosome eggs indicated by black arrows. Scale bars = 100 µm.

Infected livers and small intestines were excised and thoroughly washed with 0.1 M phosphate-buffered saline pH 7.4 (PBS), then snap-frozen in pre-cooled isopentane (Sigma-Aldrich, M32631) in liquid nitrogen. Cryosections of 20 μm thickness were prepared using a CM3050 S Research Cryostat (Leica) and mounted onto membrane frame slides (MMI, 50102). The slides were stored at –80 °C until use. To visualize tissue morphology across different phases of infection, representative sections from both the acute and chronic phases were fixed for 15 min in 4% PFA, washed in PBS, and stained with hematoxylin and eosin (**Figure 1**). These sections were not used for proteomic analysis but served for orientation in the tissues and for assessment of the histopathological changes in the tissue surrounding parasite eggs. For proteomic analysis, five biological samples were analyzed per tissue (liver and intestine) and infection phase (acute – 7 weeks, chronic – 12 weeks post-infection), resulting in a total of 20 samples.

### 2.3 Laser microdissection

**Figure 2:**
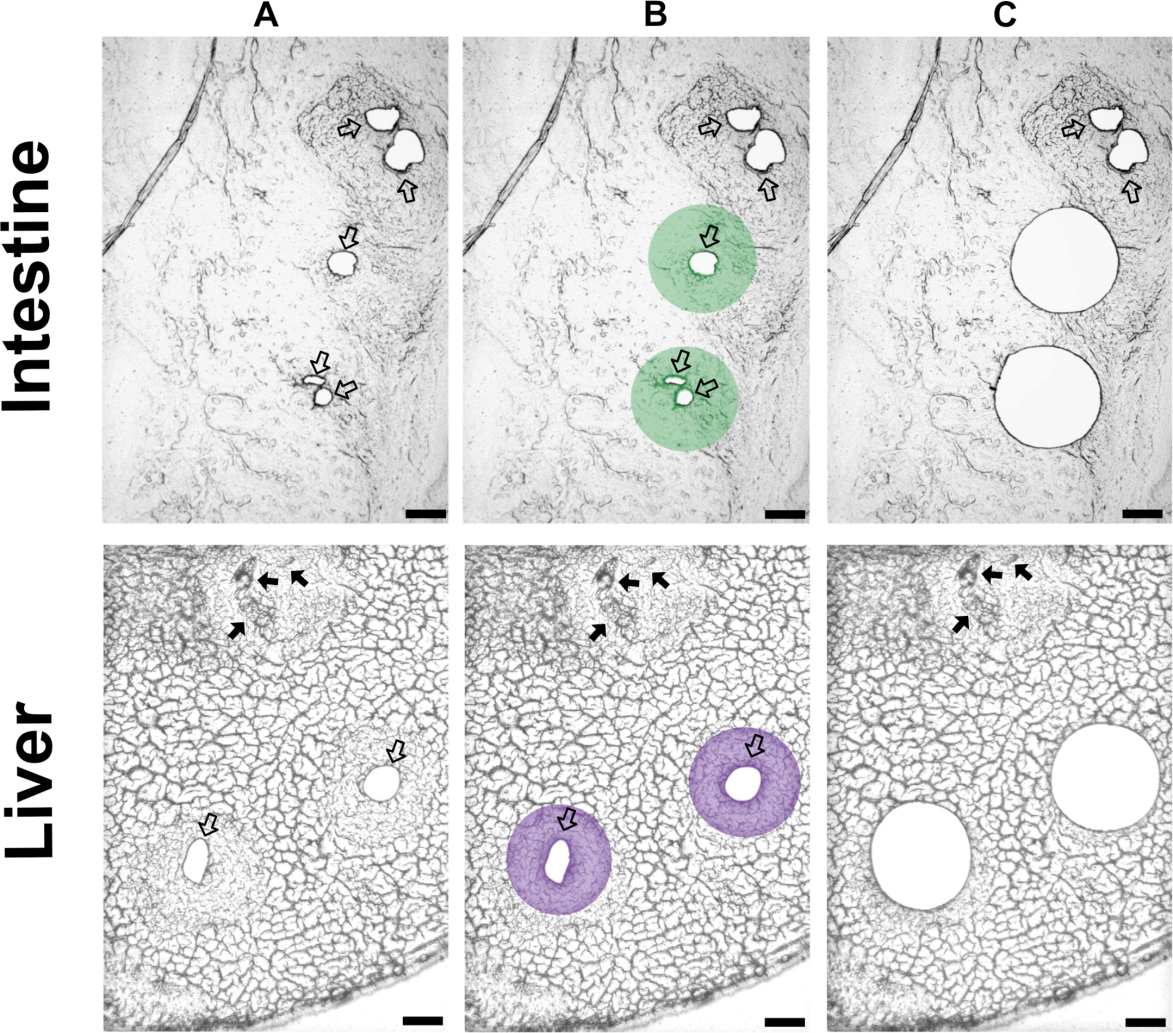
Laser microdissection of the host tissue around *Schistosoma mansoni* eggs. The figure shows cryosections (20 µm) of mouse small intestine and liver tissue. **(A)** The tissues after removal of *S. mansoni* eggs (empty spaces; transparent arrows). **(B)** The region of interest is within 100 µm around the eggs. **(C)** The microdissected tissue around the eggs was used for the proteomic analysis. Black arrows indicate schistosome eggs, while transparent arrows mark excised eggs. Scale bar = 100 µm.

Prior to microdissection, membrane slides were equilibrated to room temperature for 10 minutes. *S. mansoni* eggs found in the intestinal and liver sections were randomly selected, excised, and discarded to prevent *post-hoc* contamination of periovular tissue (**Figure 2A**). Then, the desired amount (see below) of the periovular tissue within 100 μm around the already excised egg was microdissected (**Figures 2B, C**) and subjected to proteomic analysis. Microdissections were performed using the LMD7 laser microdissection microscope (Leica) under a 10× magnification objective. Microdissected samples were collected directly into 0.5 ml plastic tubes and stored at –80 °C until mass spectrometry analysis.

The required amount of microdissected tissue, ensuring sufficient resolution and detection sensitivity, was determined based on our analysis of 10, 25, and 50 × 10^6^ μm^3^ of the periovular tissue from the liver samples. Based on proteomic analysis of two biological replicates per tissue amount (**Supplementary Figure 1**), the final decision was to analyze 25 × 10^6^ μm^3^ of tissue in each experimental sample.

### 2.4 In-solution digestion protocol

Microdissected mouse tissues were lysed by boiling at 95 °C for 10 minutes in a lysis buffer composed of 100 mM triethylammonium bicarbonate (Thermo Fisher Scientific, 17902), 2% sodium deoxycholate (Merck), 40 mM chloroacetamide (Merck, 89905), and 10 mM Tris(2-carboxyethyl)phosphine hydrochloride (Merck, 75259). Samples were subsequently sonicated using a Bandelin Sonopuls Mini 20 (MS 1.5). Proteins were digested overnight at 37 °C with 0.5 µg of MS-grade trypsin (Thermo Fisher Scientific, 90057) per sample. Following digestion, samples were acidified with trifluoroacetic acid (Merck, 80457) to a final concentration of 1% and washed three times with ethyl acetate. After evaporation of ethyl acetate, samples were re-acidified with trifluoroacetic acid, and peptides were desalted using in-house packed stage tips containing C18 Empore disks (3M), following the routine protocol (Rappsilber et al., 2007).

### 2.5 Nanoflow liquid chromatography-tandem mass spectrometry analysis

LC–MS analysis was conducted using a nano reversed-phase column (Aurora Ultimate TS, 25 × 75 µm, C18 UHPLC column; Ion Opticks). Mobile phase A consisted of 0.1% formic acid in water, and mobile phase B of 0.1% formic acid in acetonitrile. Samples were first loaded onto a trap column (C18 PepMap100, 5 µm particle size, 300 µm × 5 mm; Thermo Scientific) for 4 minutes at a flow rate of 18 µL/min using a loading buffer containing water, 2% acetonitrile, and 0.1% trifluoroacetic acid. Peptides were subsequently eluted using a gradient from 4% to 35% of mobile phase B over 60 minutes. Eluting peptide cations were ionized by electrospray and analyzed on the Orbitrap Astral mass spectrometer (Thermo Fisher Scientific) using a data-independent acquisition approach. Survey scans of peptide precursors (m/z 350–1400) were acquired in the Thermo Orbitrap Fusion device (Q-OT-qIT, Thermo Fisher Scientific) at a resolution of 60,000 (at m/z 200) with an ion target of 4 × 10^5^. Data-independent acquisition scans were acquired at 30,000 resolution with the AGC target set to 1000% and maximum injection time set to Auto. The precursor mass range from m/z 400 to 1000 was divided into 30 windows, each 20 Da wide. Higher energy collisional dissociation was used for fragmentation with a normalized collision energy of 28%.

### 2.6 MS data analysis

All data were analyzed and quantified using Spectronaut 19 (Bruderer et al., 2015) with the directDIA analysis. The data were searched against combined *S.mansoni* and *Mus musculus* protein databases. The *S. mansoni* database (translated genome, schistosoma_mansoni.PRJEA36577.WBPS19 (Coghlan et al., 2019)) was downloaded from WormBase Parasite (https://parasite.wormbase.org (Howe et al., 2017)), while the *M. musculus* database was obtained from UniProt (21701 entries). Peptides matching mouse proteins were excluded, and only peptides uniquely mapping to *S. mansoni* proteins were retained for downstream analysis. Proteins of interest were subsequently manually annotated based on accession numbers and comparison with previously published datasets (Peterková et al., 2024; Konečný et al., 2025). Enzyme specificity was defined as cleavage C-terminal to arginine and lysine residues, including cleavage at proline bonds, allowing for up to two missed cleavages. Carbamidomethylation of cysteines was set as a fixed modification, while N-terminal protein acetylation and methionine oxidation were specified as variable modifications. The false discovery rate was controlled at 1% for peptide-spectrum matches, peptides, and proteins. Only proteins supported by at least two unique peptides were considered for further analysis. Quantification was performed at the MS2 level. The precursor posterior error probability cutoff, as well as precursor and protein score thresholds, were set at 0.01. Protein-level posterior error probability was set at 0.05. Processed data were subsequently exported and analyzed in Perseus (v. 1.6.15.0) (Tyanova et al., 2016).

### 2.7 Statistical analysis and data visualization

Statistical significance was determined using a two-sample t-test in Perseus (v. 1.6.15.0) combined with permutation-based false discovery rate (FDR) correction (250 randomizations, FDR = 0.05). Proteins passing the FDR threshold were considered significantly more or less abundant statistically significant. Fold change (log_2_FC) values were calculated as log_2_-transformed ratios of normalized expression between infection phases and are presented for descriptive and visualization purposes only. PCA was performed by ClustVis (https://biit.cs.ut.ee/clustvis (Metsalu and Vilo, 2015)). Venny (v. 2.1, https://bioinfogp.cnb.csic.es/tools/venny) was used to create the Venn diagram. Final figures (including volcano plots) were created using GraphPad Prism (v. 10.6.1) and Affinity Photo (v. 2.0).

## 3 Results

### 3.1 Histological confirmation of granuloma architecture

First, mouse liver and small intestinal tissues from both the acute and chronic phases of infection were cryosectioned and stained with hematoxylin and eosin to assess tissue integrity and granuloma development. We confirmed that granuloma formation was maintained, and the observed pathology corresponded to the phase of infection (Amaral et al., 2017). Specifically, exudative-productive granulomas with viable inflammatory cells and fine collagen fibers concentrated around the eggs were most commonly found (**Figure 1**). This evaluation confirmed that the tissues were suitable for microdissection and proteomic profiling, which was further performed on unstained cryosections.

### 3.2 Optimization of laser microdissection sampling for proteomic analysis

To determine the optimal amount of microdissected tissue for proteomic analysis (to ensure optimal detection of parasite proteins), we compared protein recovery from liver granuloma samples of 10, 25, and 50 × 10^6^ µm^3^. Randomly selected eggs were excised and removed prior to tissue collection to prevent contamination (**Figure 2A**). The surrounding host tissue within a 100 µm radius of the original egg position was then isolated by laser microdissection (**Figures 2B, C**). This strategy enabled the collection of host tissue directly exposed to egg excretions/secretions while excluding the egg itself. Proteomic analysis revealed that on average, 110, 174, and 158 *S. mansoni* proteins were detected in these respective volumes, corresponding to 2.3%, 3.0%, and 2.7% of the total proteome detected (**Supplementary Figure 1**). Although the largest volume yielded more total protein, it did not result in a further increase in the number of parasite-specific proteins. Therefore, the volume of 25 × 10^6^ µm^3^ was selected as the optimal sample volume for subsequent proteomic analyses.

### 3.3 Reproducibility of *in situ* egg-associated proteomes across biological replicates

**Figure 3:**
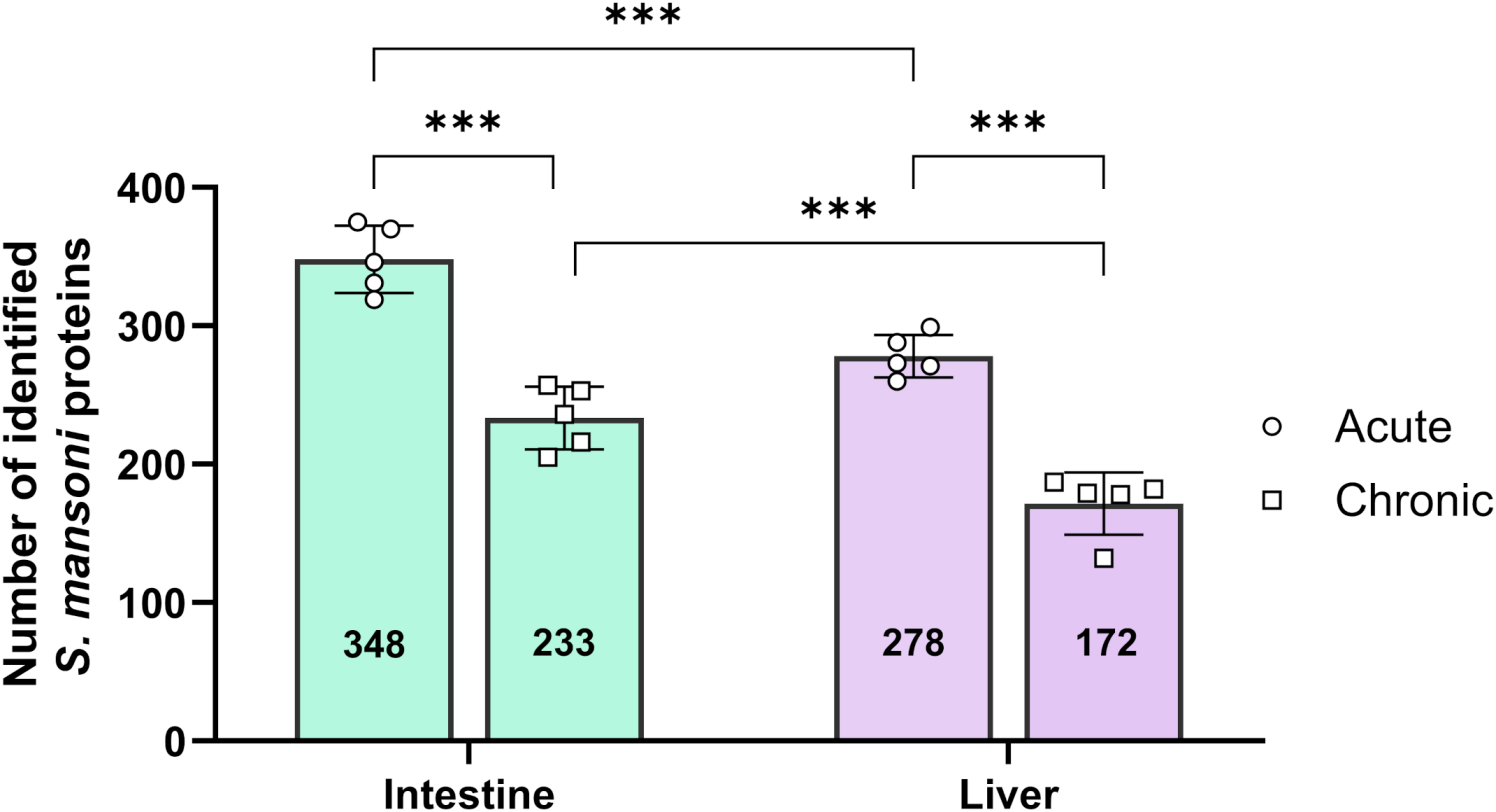
Quantification of *Schistosoma mansoni* proteins detected in the microdissected mouse tissues. Number of proteins identified in small intestine and liver tissues (25 × 10^6^ µm^3^ per sample); proteins were counted if detected in at least 3 out of 5 replicates. Points show data from individual mice; bars show mean ± SD. Statistical significance was determined by 2-way ANOVA and Holm-Šídák test (*** p-value < 0.001). Bold values indicate the mean number of schistosomal proteins detected in each tissue type.

Using this standardized microdissection and sample preparation strategy, unfixed and unstained cryosections of liver and small intestinal tissues from five biological replicates (mice) per infection phase (acute and chronic) were processed. For quantitative analyses, only proteins detected in at least three biological replicates were retained to ensure data robustness. In the small intestine, a mean of 348 *S. mansoni* proteins (3.8% of the total proteome) was detected during the acute phase, decreasing to 233 proteins (2.7%) in the chronic phase. A similar pattern was observed in the liver, where a mean of 278 schistosome-derived proteins (3.2%) was detected in the acute phase compared with 172 proteins (2.1%) in the chronic phase. Thus, the number of detectable *S. mansoni* proteins declined consistently from acute to chronic infection (p<0.001) and was significantly affected by the tissue origin (p<0.001) (**Figure 3**; **Supplementary Data 1**).

### 3.4 Phase-specific segregation of periovular proteomes

**Figure 4:**
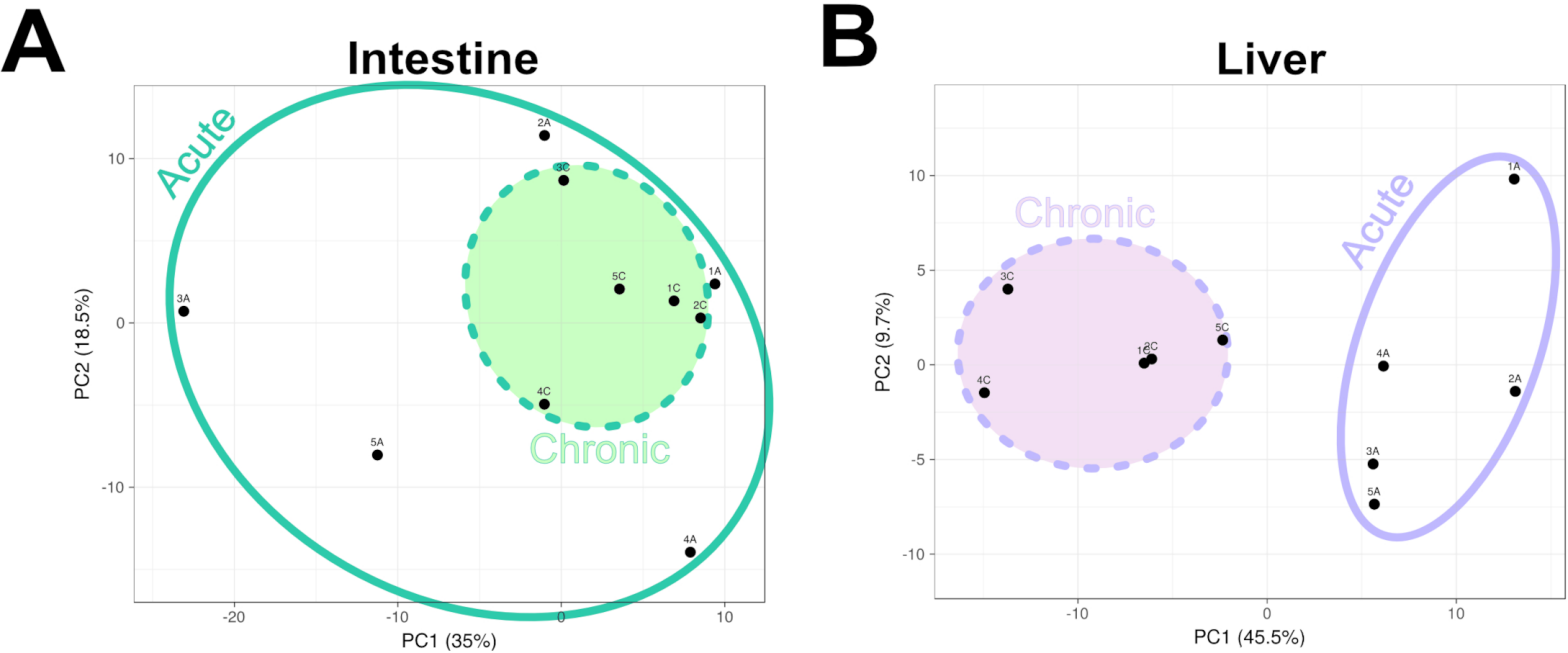
Principal component analysis (PCA) of intestinal and liver proteome profiles identifying *Schistosoma mansoni* proteins within host tissues. **(A)** PCA of intestinal samples showing partial separation between acute and chronic infection phases. **(B)** PCA of liver samples showing clear separation between acute and chronic infection phases.

Principal component analysis (PCA) revealed distinct patterns of proteomic variation between the intestine and liver (**Figure 4**; **Supplementary Data 2**). In intestinal tissues (**Figure 4A**), acute and chronic samples showed only partial separation, indicating that the intestinal proteome remained relatively similar across infection phases. Chronic samples clustered tightly, suggesting a more uniform proteomic profile. In contrast, acute intestinal samples displayed greater variability. A subset of acute replicates (samples 1A and 2A in **Figure 4A**) localized near the chronic cluster, whereas others (e.g., sample 3A in **Figure 4A**) exhibited a signature more distinct from the acute phase cluster. In liver tissues (**Figure 4B**), however, acute and chronic samples formed two well-separated, non-overlapping clusters, indicating more pronounced and consistent proteomic differences between the infection phases. This more pronounced separation suggests that the proteomic profiles of liver granulomas differ more significantly between the acute and chronic phases than those of intestinal granulomas.

### 3.5 A conserved core egg excretome/secretome is shared across tissues and infection phases

**Figure 5:**
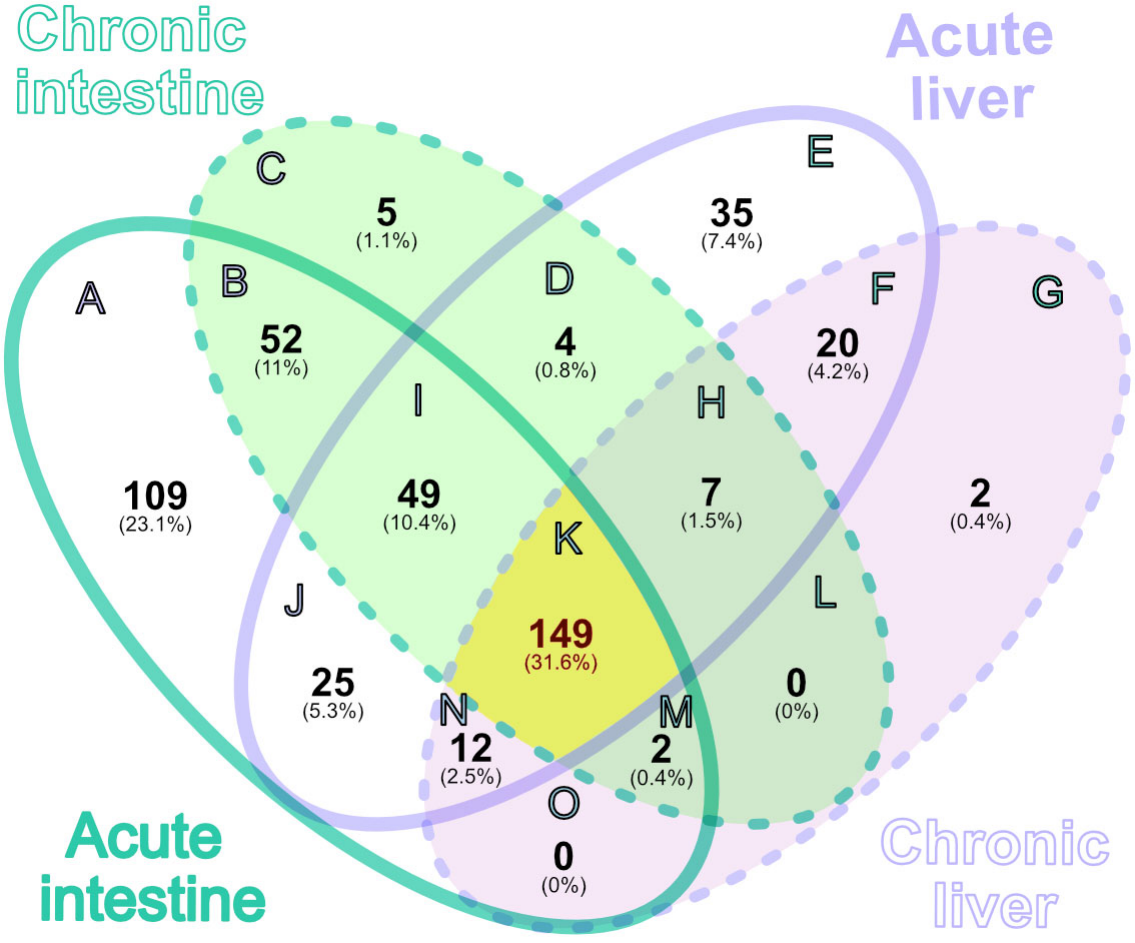
Venn diagram showing overlap of identified proteins across four groups (acute/chronic intestine, acute/chronic liver). Numbers denote counts of proteins detected in each combination (presence/absence only). The yellow region highlights the 149 core proteins detected in all four groups (region K).

To further analyze *S. mansoni* proteomes in the periovular granuloma areas, we generated a four-way Venn diagram (**Figure 5**; **Supplementary Data 3**). In total, 149 schistosome proteins (31.6% of all detected parasite proteins) were detected under all four conditions (acute intestine, chronic intestine, acute liver, chronic liver), representing a conserved core proteome present across tissues and infection phases (region K). This core proteome contained most key molecules of interest, including omega-1 isoforms, IPSE/α-1, and micro-exon gene family members (MEG) and the venom allergen-like proteins (VAL). Core proteases (aminopeptidase N1, thimet oligopeptidase, dipeptidyl peptidase 3) and several inhibitors (serpin, cystatin, α2-macroglobulin) were also consistently present. A substantially larger number of proteins were uniquely detected in the acute intestine (109 proteins, 23.1%; region A), whereas the chronic intestine subset contained only 5 unique proteins (1.1%; region C). In the liver, 35 proteins (7.4%; region E) were specific to the acute phase, while only 2 proteins (0.4%; region G) were unique to the chronic phase.

Comparison across infection phases within each tissue showed that the intestine shared 52 proteins between acute and chronic phases (11%; region B), while the liver shared 20 proteins across phases (4.2%; region F). Overlap across tissues within the same infection phase revealed 25 proteins shared in the acute phase (5.3%; region J) and 0 in the chronic phase (0%; region L). Thus, the acute infection phase in both tissues is characterized by a higher proportion of unique schistosomal proteins, whereas the chronic phase exhibits a reduced and more overlapping parasite proteome.

### 3.6 Intestinal periovular proteomes change minimally between acute and chronic infections

**Figure 6:**
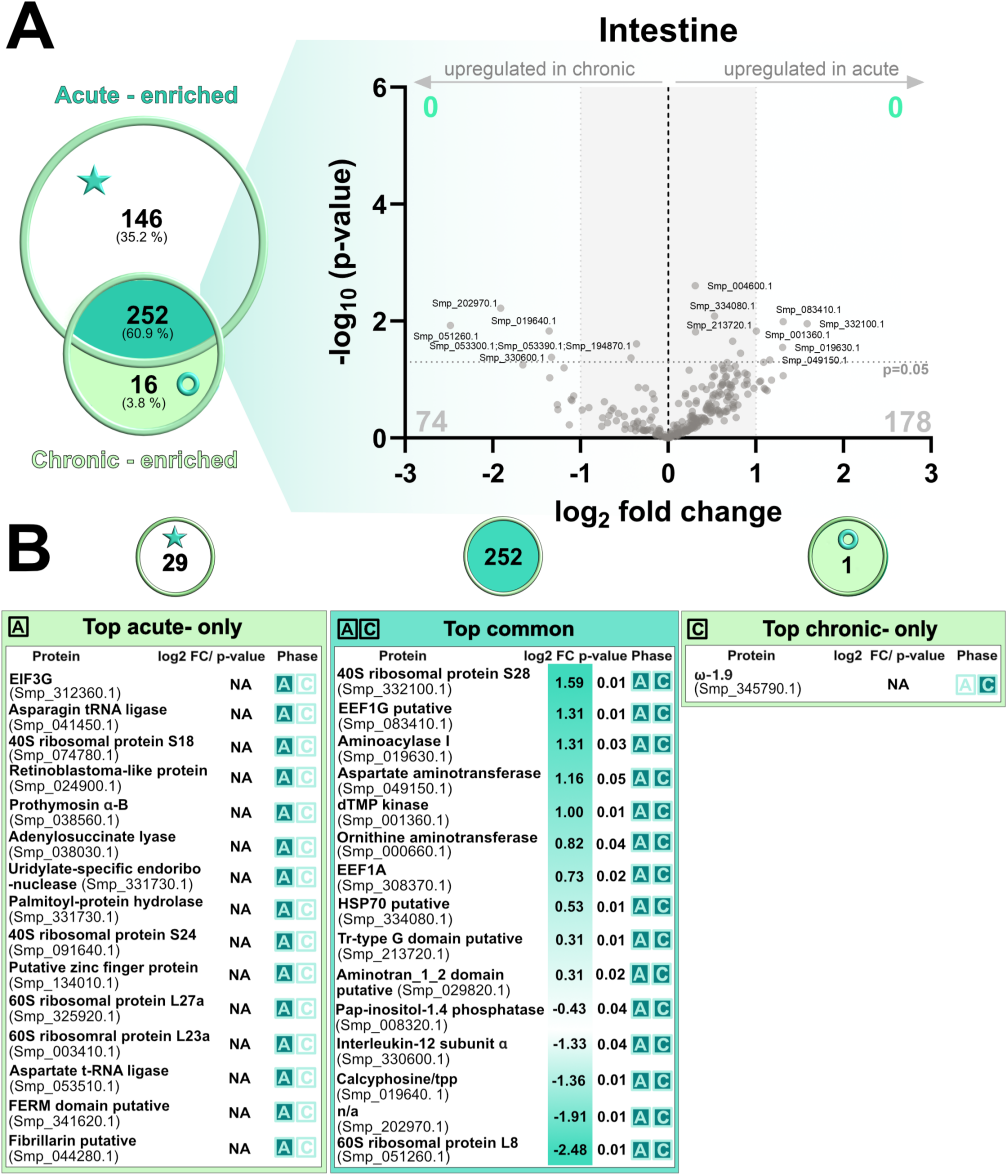
Comparative proteomic analysis of intestinal proteins during acute and chronic infection phases. **(A)** Venn diagram showing the overlap of proteins identified in the intestine during the acute and chronic infection phases. The accompanying volcano plot illustrates log_2_ fold changes (log_2_FC) and significance values (–log_10_ p-values) for intestinal proteins shared between the two conditions. Significantly more abundant proteins (after FDR adjustment) are highlighted in green, while FDR non-significant proteins are shown in grey. **(B)** tables summarizing top proteins uniquely detected in the acute phase (left), common to both phases (middle, “shared proteome”), and identified only in the chronic phase (right). For each protein, fold change (FC), p-value, and infection phase are indicated. The grey dashed horizontal line indicates the statistical significance threshold (p-value < 0.05), while the vertical black line at log_2_FC = 0 represents no change between conditions. Proteins with low log_2_FC values are shaded grey. The term “enriched” is used to describe proteins consistently detected in at least three of five biological replicates during the given phase and in no more than two replicates of the other. Proteins described as “phase-only” were detected in one phase only and were completely absent (0 replicates) in the other phase.

Proteomic analysis of intestinal samples revealed distinct protein profiles between the acute and chronic phases of infection (**Figure 6**; **Supplementary Data 4**). A total of 414 proteins were identified, of which 146 were enriched in the acute phase, 16 were enriched in the chronic phase, and 252 were present in both phases (“shared proteome”). Differential abundance analysis of the shared proteome did not identify any significantly more abundant proteins (**Figure 6**). The most differentially regulated proteins present in the shared proteome (“top common”) were primarily associated with core cellular processes, including protein synthesis (elongation factor 1α (EEF1A), elongation factor 1γ (EEF1G), 40S ribosomal protein S28, 60S ribosomal protein L8), amino acid and nucleotide metabolism (ornithine aminotransferase, aspartate aminotransferase, dTMP kinase), and cellular signaling and regulation (interleukin-12 subunit α, calcyphosine/TPP, T-complex protein 1 subunit γ, pap-inositol-1,4-phosphatase).

Proteins exclusively detected only in the acute phase (29 proteins) of the intestine were primarily associated with protein synthesis, including ribosomal subunits (40S S18 and S24; 60S L27a and L23a), translation elongation factors, and tRNA ligases. We also observed several proteins involved in gene regulation (retinoblastoma-like protein, zinc finger protein, prothymosin α, and an uridylate-specific endoribonuclease). In addition, proteins involved in nucleotide metabolism (adenylosuccinate lyase) and membrane organization (palmitoyl-protein hydrolase, FERM domain protein) were upregulated. Only one protein was uniquely detected in the intestinal chronic phase, identified as omega-1.9, indicating that phase-specific protein excretion/secretion is negligible during chronic infection compared to the acute phase. Overall, these findings suggest that although certain excreted/secreted proteins vary between infection phases, the core intestinal proteome remains relatively stable.

### 3.7 Liver periovular proteomes undergo marked remodeling during chronicity

**Figure 7:**
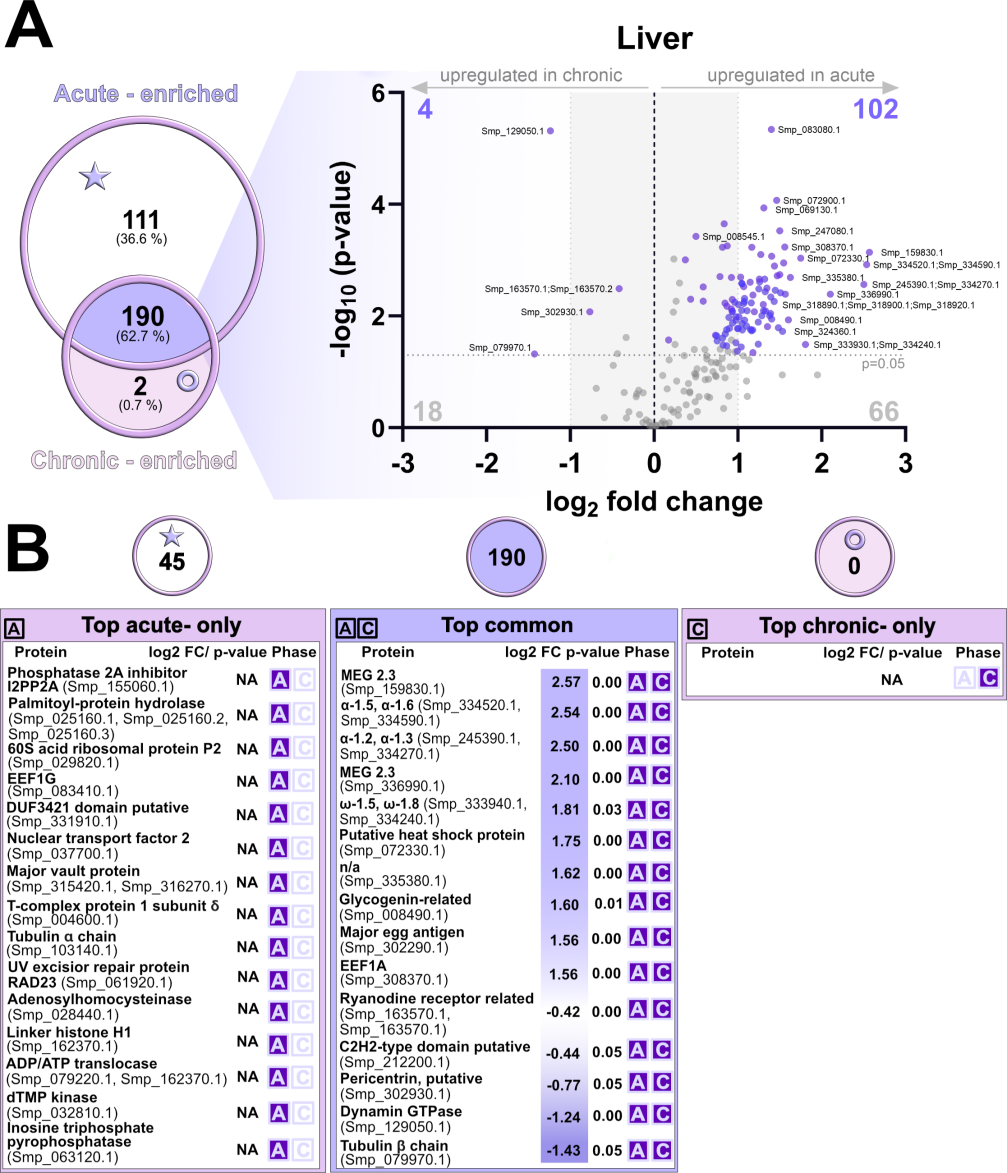
Comparative proteomic analysis of liver proteins during acute and chronic infection phases. **(A)** Venn diagram showing the overlap of proteins identified in the liver during the acute and chronic infection phases. The accompanying volcano plot illustrates log_2_ fold changes (log_2_FC) and significance values (–log_10_ p-values) for liver proteins shared between the two conditions. Significantly more abundant proteins (after FDR adjustment) are highlighted in violet, while FDR non-significant proteins are shown in grey. **(B)** Tables summarizing top proteins uniquely detected in the acute phase (left), common to both phases (middle, “shared proteome”), and identified only in the chronic phase (right). For each protein, fold change (FC), p-value, and infection phase are indicated. The grey dashed horizontal line indicates the statistical significance threshold (p-value < 0.05), while the vertical black line at log_2_FC = 0 represents no change between conditions. Proteins with low log_2_FC values are shaded grey. The term “enriched” is used to describe proteins consistently detected in at least three of five biological replicates during one infection phase and in no more than two replicates of the other. Proteins described as “phase-only” were detected in one phase only and were completely absent (0 replicates) in the other phase.

Proteomic profiling of liver tissue revealed marked differences between the acute and chronic phases of infection, in contrast to the relatively stable intestinal egg excretome/secretome (**Figure 7**; **Supplementary Data 5**). A total of 303 proteins were identified, including 111 enriched in the acute phase, 2 enriched in the chronic phase, and 190 shared between both phases (referred here as “shared proteome”). Among these shared proteins, 102 proteins were significantly more abundant in the acute phase and 4 proteins in the chronic phase (**Figure 7**). The most differentially regulated proteins present in the shared proteome (“top common” group) were dominated by micro-exon gene (MEG) family members, together with IPSE/α-1 and omega-1 isoforms, well-known immunomodulatory effectors released by eggs during granuloma formation. In addition, several stress response and protein-folding proteins (e.g., HSP16, HSP20, HSP90 co-chaperone, and 14-3–3 proteins) were found to be more abundant in top common group. Major egg antigens, along with glycogenin-related enzymes, aminopeptidases, and EEF1A, were also significantly more abundant, reflecting active protein synthesis, nutrient processing, and sustained excretory/secretory activity at this phase. In contrast, shared proteins significantly more abundant during the chronic phase were primarily linked to cellular structure and regulatory signaling, including a ryanodine receptor-related protein (calcium signaling), a C2H2-type transcription factor, pericentrin and tubulin β-chain (microtubule organization), and dynamin GTPase involved in membrane remodeling and vesicle trafficking.

Proteins detected exclusively in the acute phase (45 proteins) of infection were involved in the translation (60S acidic ribosomal protein P2, EEF1G, and T-complex protein 1 subunit δ), nucleotide metabolism (dTMP kinase, ITPase, adenosylhomocysteinase), and cellular transport and structural regulation (vault protein, NTF2, tubulin α, histone H1). In contrast, no proteins were uniquely detected in the chronic phase.

### 3.8 Acute liver granulomas are enriched for key immunomodulatory and excretory/secretory egg proteins

**Figure 8:**
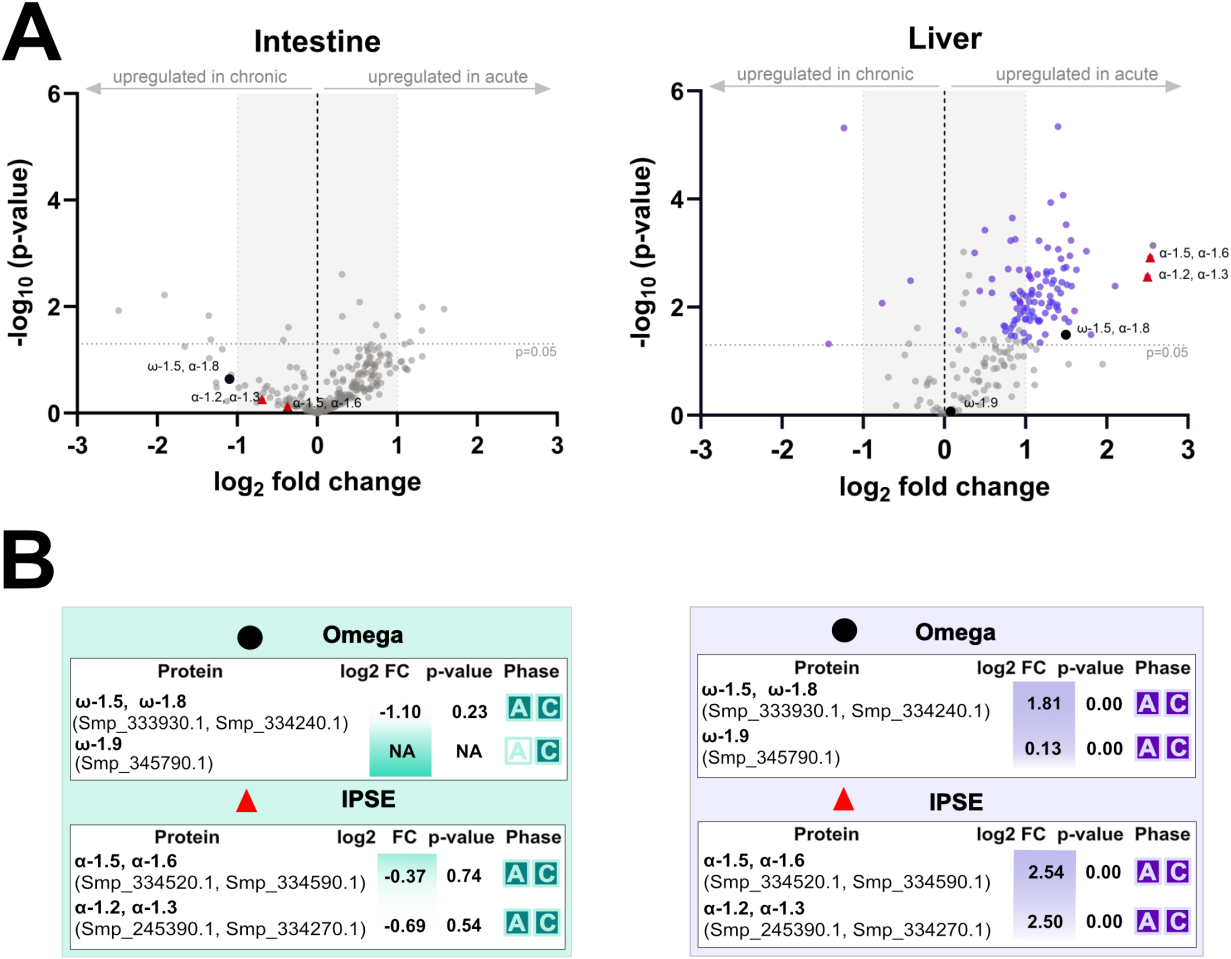
Comparative proteomic analysis of intestine and liver immunomodulatory molecules during acute and chronic infection phases. **(A)** volcano plots illustrate log2 changes (log_2_FC) versus statistical significance (–log_10_ p-values) of schistosomal immunomodulatory proteins (omega-1, IPSE/α-1 isoforms) detected in microdissected intestine (left) and liver (right) tissues surrounding schistosome eggs. The grey dashed horizontal line indicates the statistical significance threshold (p-value < 0.05), while the vertical black line at log_2_FC = 0 represents no change between conditions. Proteins with low log_2_FC values are shaded grey. **(B)** the tables below summarize protein identities, log_2_FC, p-values, and the infection phase in which they were detected (A = acute, C = chronic).

**Figure 9:**
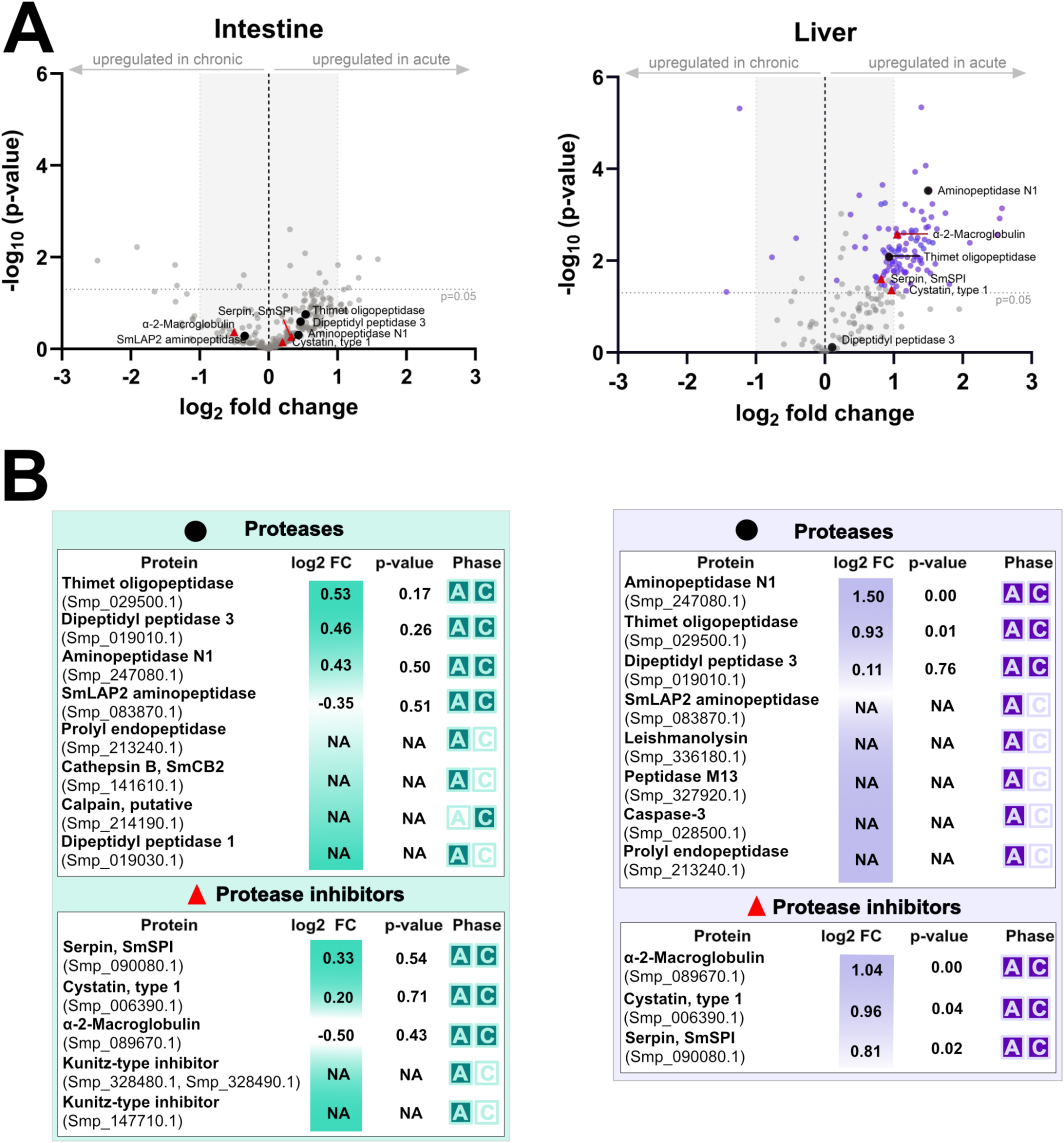
Comparative proteomic analysis of intestine and liver proteases and inhibitors during acute and chronic infection phases. **(A)** volcano plots illustrate log_2_ fold changes (log_2_FC) versus statistical significance (–log_10_ p-values) of schistosomal proteases and inhibitors detected in microdissected intestine (left) and liver (right) tissues surrounding schistosome eggs. The grey dashed horizontal line indicates the statistical significance threshold (p-value < 0.05), while the vertical black line at log_2_FC = 0 represents no change between conditions. Proteins with low log_2_FC values are shaded grey. **(B)** the tables below summarize protein identities, log_2_FC, p-values, and the infection phase in which they were detected (A = acute, C = chronic).

**Figure 10:**
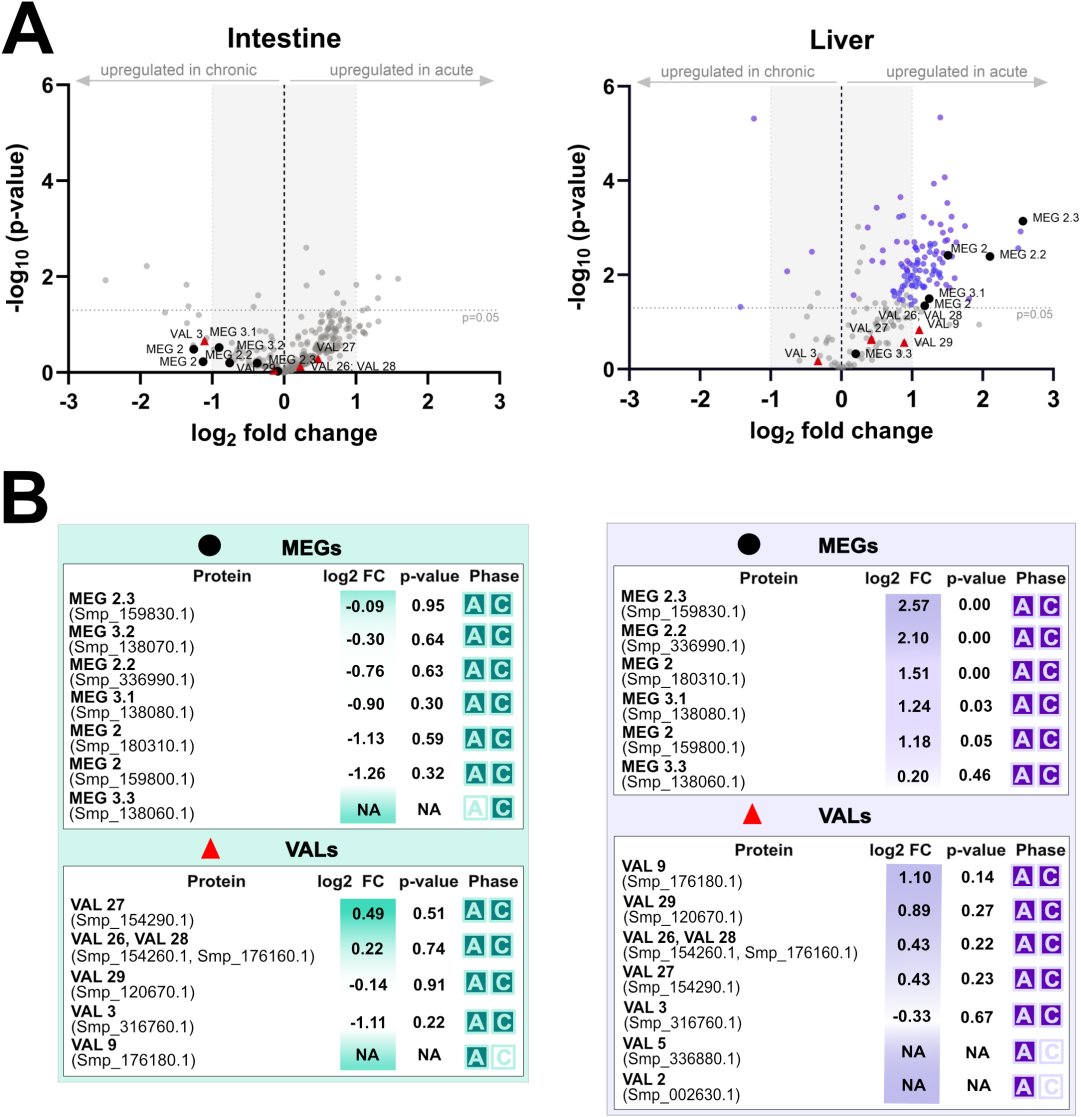
Comparative proteomic analysis of intestine and liver MEGs and VALs during acute and chronic infection phases. **(A)** volcano plots illustrate log_2_ fold changes (log_2_FC) versus statistical significance (–log_10_ p-values) of schistosomal MEGs and VALs protein detected in microdissected intestine (left) and liver (right) tissues surrounding schistosome eggs. The grey dashed horizontal line indicates the statistical significance threshold (p-value < 0.05), while the vertical black line at log_2_FC = 0 represents no change between conditions. Proteins with low log_2_FC values are shaded grey. **(B)** the tables below summarize protein identities, log_2_FC, p-values, and the infection phase in which they were detected (A = acute, C = chronic).

**Figure 11:**
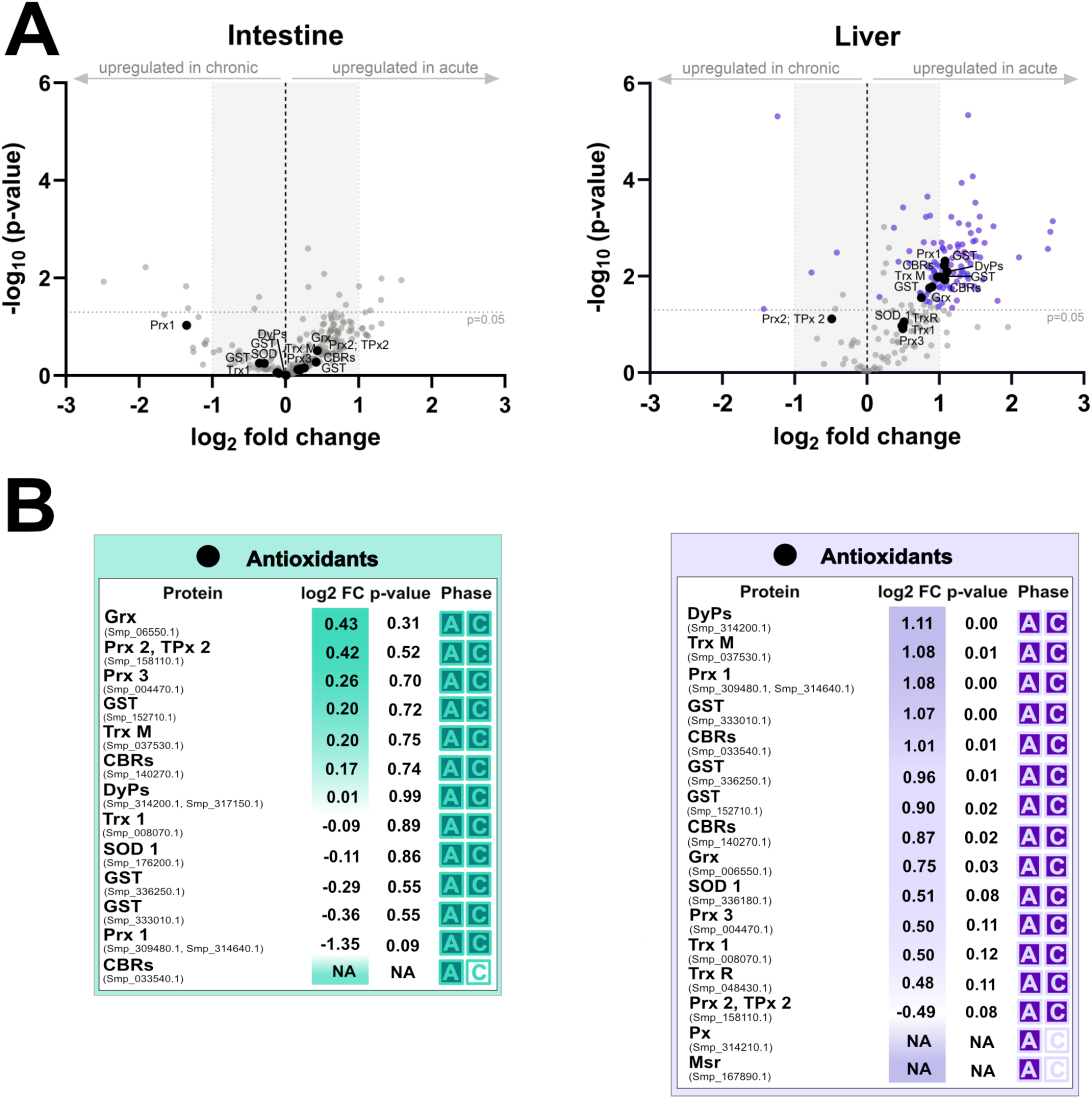
Comparative proteomic analysis of intestine and liver antioxidants during acute and chronic infection phases. **(A)** volcano plots illustrate log_2_ fold changes (log_2_FC) versus statistical significance (–log_10_ p-values) of schistosomal antioxidative proteins detected in microdissected intestine (left) and liver (right) tissues surrounding schistosome eggs. The grey dashed horizontal line indicates the statistical significance threshold (p-value < 0.05), while the vertical black line at log_2_FC = 0 represents no change between conditions. Proteins with low log_2_FC values are shaded grey. **(B)** the tables below summarize protein identities, log_2_FC, p-values, and the infection phase in which they were detected (A = acute, C = chronic).

Analysis of microdissected tissues surrounding *S. mansoni* eggs revealed a distinct, tissue- and phase-specific pattern in the excretion/secretion of parasite proteins (**Supplementary Data 6**, **7**). In all four functional groups examined – immunomodulatory molecules (**Figure 8**), proteases and protease inhibitors (**Figure 9**), MEGs/VALs proteins (**Figure 10**), and antioxidant molecules (**Figure 11**) – we observed significantly more abundant proteins in the acute liver samples, while the amount of proteins in the intestinal samples, regardless the phase, remained unaltered and did not reach statistical significance. These findings indicate that *S. mansoni* proteins of those particular groups detected in periovular areas are preferentially enriched in hepatic granulomas during the acute phase of infection. On the contrary, intestinal periovular areas show no significant changes in protein abundance over the phases of infection.

## 4 Discussion

Molecules released from *S. mansoni* eggs play a vital role in mediating host-parasite interactions, including the immune modulation and related tissue pathology. Proteomic analyses of these molecules have traditionally relied on collecting ESPs from eggs cultivated *in vitro*, generating important catalogs of potential immunomodulators, antigens and enzymes (Cass et al., 2007; Mathieson and Wilson, 2010; Dvořák et al., 2016; Carson et al., 2020). However, substantial quantitative and qualitative variability across these studies have left open the question of which parasite proteins genuinely interact with the host under physiological conditions, and which may represent artifacts of the experimental systems. In this study, our tissue-centric laser microdissection approach enabled us to provide a tissue-contextualized “snapshot” of parasite-derived proteins within the periovular granulomas in the mouse liver and small intestine during both acute and chronic phases of infection. Hence, we provide the true *in situ* excretome/secretome of *S. mansoni* eggs, directly reflecting the molecular interaction within the host.

Across both microdissected tissues and phases of infection, we consistently detected 149 schistosomal proteins, representing a conservative “core” excretome/secretome likely essential for egg survival and host interactions. This number aligns more closely with the first egg ESP study, which reported 188 proteins (Cass et al., 2007), in contrast to a later study that identified only 6 proteins from liver eggs during the acute phase (Mathieson and Wilson, 2010). Although the detection of such a high number of proteins, including canonical intracellular proteins such as folding factors, structural proteins, and glycolytic enzymes, has traditionally been attributed to egg damage or death during isolation and *in vitro* culture, our data show that these molecules are indeed present at the host-parasite interface. This finding aligns well with the growing recognition that many of these typically intracellular proteins act as “moonlighting” proteins across various pathogens, also playing extracellular roles in host-parasite interactions, such as immunomodulation or tissue remodeling (Gómez-Arreaza et al., 2014; Jeffery and Jeffery, 2019; Ancarola et al., 2023). Regardless of whether these proteins are actively excreted/secreted or released due to egg damage within tissues, our findings provide a physiological snapshot of reality, demonstrating that periovular tissues in the liver and intestine encounter a complex repertoire of parasite-derived proteins during schistosome infection. This confidence is further supported by our strict data filtering, which considers only proteins consistently detected across biological replicates, and the removal of somatic egg material prior to collecting the target periovular tissue.

A striking result from our analyses is the pronounced difference in the dynamics of egg-excreted/secreted proteins in acute and chronic phases in the liver and intestine. Across both tissues, the number of detected schistosomal proteins decreased significantly from acute to chronic infection; however, the liver eggs showed a far stronger separation between acute and chronic excretomes/secretomes (clear PCA clustering) and a greater number of proteins uniquely detected or significantly upregulated in the acute phase. Importantly, canonical egg immunomodulators IPSE/α-1 and omega-1 were significantly upregulated in hepatic periovular tissue during acute infection. This is consistent with the liver being a dominant site where egg-derived immunomodulation shapes granulomas and systemic immunity soon after oviposition, aligning with our previous findings showing preferential gene expression of these immunomodulators in the liver eggs (Acharya et al., 2021; Peterková et al., 2024). It appears that the prominent presence of these immunomodulators in the liver, promoting Th2 polarization (omega-1) and IL-4-dependent alternative activation of macrophages (IPSE/α-1), contributes to limiting tissue injury and controlling hepatic inflammation. The Th2 immune response, IL-4, and alternatively activated macrophages are indeed critical for protection against organ injury during schistosomiasis (Brunet et al., 1997; Herbert et al., 2004) and other infections with tissue-dwelling helminths (Chen et al., 2012; Gause et al., 2013). Hence, we propose that the abundant presence of omega-1 and IPSE/α-1 at the parasite-liver interface represents a co-adapted strategy to maintain host tissue integrity during deposition of eggs in the liver, which shows higher egg loads over the long term compared to the eggs in the intestine (Montasser et al., 2024; Peterková et al., 2024). This concept also complements the hypothesis that IPSE/α-1 mitigates inflammation potentially triggered by microbial translocation during egg passage through the intestinal wall (Knuhr et al., 2018), supporting a context-dependent protective function across multiple tissues.

In contrast, the intestinal egg excretome/secretome showed comparatively modest qualitative differences between acute and chronic phases. Although the number of egg-excreted/secreted proteins decreased substantially from acute to chronic infection, indicating a clear quantitative shift, intestinal samples show more uniform excretion/secretion across phases, resulting in overlapping phase clusters. Based on this result, it is tempting to speculate on the nature of intestinal granulomas in the chronic phase. Although it has been implied that acute and chronic granulomas differ in the intestine (Schwartz and Fallon, 2018), there is limited clear evidence for distinct intestinal granuloma stages. Whereas the temporal development of hepatic inflammation and granuloma is well characterized (Amaral et al., 2017; Costain et al., 2022), including defined transitions through pre-granulomatous exudative, necrotic-exudative, exudative-productive, and productive stages, intestinal granulomas appear less variable over time. Small-intestinal granulomas predominantly present solely as exudative-productive structures, without the full progression observed in the liver (Amaral et al., 2017). This relative homogeneity is further supported by data showing that granuloma size, a proxy for inflammation intensity, remains remarkably stable throughout infection in the intestine (Weinstock and Boros, 1981, 1983; Silva et al., 2000; Turner et al., 2012). Together, these observations suggest that acute and chronic small-intestinal granulomas may be more similar to each other than their hepatic counterparts, consistent with the relatively modest differences we observed in the intestinal egg excretome/secretome.

The distinct biology of egg deposition in the liver versus the intestine likely underlies the differences observed in their protein excretion/secretion. Hepatic eggs become permanently trapped and progressively remodel their surrounding niche, resulting in a fully differentiated chronic granulomatous state. By contrast, most intestinal eggs traverse the tissue over a short, well-defined window of approximately 6 days (Schwartz and Fallon, 2018). Therefore, the excreted/secreted proteins detected in the intestine during both the acute and chronic phases could likely arise mostly from newly deposited eggs. This interpretation fits our data: intestinal eggs in chronic infection excrete/secrete fewer proteins overall, yet maintain a qualitatively similar excretome/secretome compared to the acute phase. These findings emphasize that the operational definitions of “acute” and “chronic” phases, based solely on weeks post-infection, do not apply equally across tissues. In the liver, chronicity reflects the accumulation and long-term persistence of egg-induced granulomatous lesions within a confined tissue microenvironment, where newly trapped eggs continue to be added to pre-existing pathology. In the intestine, however, chronicity reflects prolonged infection of the host, whereas the eggs present at any given time are predominantly newly laid and thus functionally resemble an acute phase. This distinction is reinforced by recent transcriptomic and phenotypic evidence demonstrating tissue-specific differences between liver- and intestine-derived eggs and miracidia (Peterková et al., 2024; Willett et al., 2025).

This distinction has practical implications. As most previous egg-focused studies relied on liver-derived eggs, they may have disproportionately reflected liver-specific immune-related egg biology and overlooked the major players functioning in intestinal tissues, which are critical for passage and transmission. Taken together, the fact that the observed differences between eggs in the acute phase, and even more so in the chronic phase, can be largely explained by the “age” of the eggs themselves may be seen as a limitation. In addition, differences between acute and chronic phases of infection may also reflect variation in egg viability and developmental stage. In chronic infection, a higher proportion of eggs may be deteriorating or developmentally impaired, which could influence the production and/or release of proteins. Therefore, the observed decrease in these proteins in chronic liver tissue may not solely reflect regulated changes in secretion, but also differences in the physiological condition of the eggs. However, we see it as a major discovery, as our data provide a true snapshot of *in situ* reality across different infection phases and are not a direct comparison between organs.

Another noteworthy result is the consistent detection of members of MEG family and VAL (SmVAL) proteins in periovular tissues. MEG proteins have long been proposed as potential egg-secreted immunomodulators based on their unique gene structure, expression profiles, and prior proteomic evidence (DeMarco et al., 2010; Mathieson and Wilson, 2010). In our dataset, a relatively stable palette of MEGs was detected across both organs and infection phases, with a particularly strong representation in the periovular liver tissues during the acute phase. This pattern mirrors the expression and secretion dynamics of the canonical immunomodulators IPSE/α-1 and omega-1. Such a parallel strongly supports the hypothesis, which needs to be experimentally tested, that MEGs may play a notable role in shaping the early immune environment at the site of oviposition (DeMarco et al., 2010).

VALs have also long been considered important players in modulating the host throughout the life cycle of not only schistosomes but also many other helminths, yet their role in the biology of the *S. mansoni* egg remains unclear (Yoshino et al., 2014; Wilbers et al., 2018; Lu et al., 2021). In our recent revision of the SmVAL family, we were unable to demonstrate the secretion of highly expressed SmVAL9 and SmVAL29 into host tissues from eggs; therefore, we assumed that egg-specific SmVALs are miracidial proteins in ovo. This conclusion was supported by their localization in the penetration glands and on the surface of the invasive larva, as well as by their lack of interaction with a range of human cell receptors (Attenborough et al., 2024; Konečný et al., 2025). However, since our current data clearly show that these and other SmVAL proteins are stably present in the tissues surrounding the eggs in both organs and phases of infection, it appears that these proteins are indeed secreted, regardless of the presence/absence of signal peptides. This observation finally confirms that SmVALs are not merely “larval-transformation” proteins, but they could still play a role in egg-host interactions.

However, VALs and MEGs are not the only proteins that often lack a classic signal peptide and yet are abundant in the vicinity of eggs. For instance, serpin (SmSPI) is not only one of the most highly expressed peptidase inhibitors in *S. mansoni* eggs but also one of the most abundant parasite proteins in the liver during the acute phase of infection. Moreover, in our separate study, we have confirmed its secretion into the host’s bile, despite the lack of a classic secretory sequence (Peterkova et al., 2023)(Konečný et al., under review). Collectively, these findings suggest a broader view than “secreted vs non-secreted” based on signal peptide, as often proposed. Proteins lacking canonical signal peptides may nonetheless reach the tissue environment via non-classical secretion, egg damage/lysis at specific sites, or association with extracellular vesicles (Cass et al., 2007; Abou-El-Naga, 2022). Therefore, the absence of a signal peptide should not automatically exclude a protein from consideration as a biologically relevant player.

Proteases and their inhibitors also emerged as key molecular groups displaying distinct, tissue-specific secretion patterns in the periovular environment. We identified a conserved core set of metalloproteases (aminopeptidase N1, thimet oligopeptidase, and dipeptidyl peptidase 3) present in both liver and intestinal tissues, with all three showing a pronounced and statistically significant increase in the liver during the acute phase of infection. This enrichment is consistent with our earlier observations, in which aminopeptidase N1 and thimet oligopeptidase were among the most highly expressed proteases in liver-derived eggs (Peterkova et al., 2023). A similar pattern was evident for protease inhibitors, which are essential in maintaining proteolytic balance. In agreement with previous egg excretome/secretome analyses and our gene expression data, cystatin type-1, serpin (SmSPI), and α-2-macroglobulin (Cass et al., 2007; Mathieson and Wilson, 2010; Carson et al., 2020; Peterkova et al., 2023; Peterková et al., 2024) represented the predominant inhibitors in both tissues and were strongly upregulated in the acute liver phase. Additionally, liver tissue shows selective enrichment of egg-specific leishmanolysin peptidase, peptidase M13, and caspase-3 – proteases implicated in extracellular matrix remodeling, inflammatory signaling, and apoptotic pathways (Huston et al., 2000; Bland et al., 2008; Olivier et al., 2012). In contrast, intestinal tissue granuloma contained exclusively cysteine proteases, including cathepsin B2 (SmCB2), calpain, and dipeptidyl peptidase 1, as well as Kunitz-type inhibitors. These findings are consistent with the literature, which demonstrates the presence of cysteine protease activities in the excretory/secretory products of *S. mansoni* eggs at neutral pH (Ashton et al., 2001). Live eggs have also been shown to degrade glycoprotein components of an artificial extracellular matrix under the same conditions, and the enhancement of this activity in the presence of a reducing agent provides further evidence for the involvement of cysteine peptidases (Pino-Heiss et al., 1985). The absence of these molecules in liver tissue, where eggs remain permanently trapped, further supports the hypothesis that eggs secrete specific cysteine proteases that actively facilitate their passage through host tissues and allow their subsequent release into the external environment via the digestive tract (Dvořák et al., 2016). This hypothesis is further supported by the presence of Kunitz-type inhibitors, which can regulate host serine proteases involved in digestion, blood coagulation, and immune responses (Shigetomi et al., 2010; Ranasinghe and McManus, 2013). These inhibitors can thus contribute to shaping the local microenvironment that facilitates the transport of the eggs.

Although our microdissection-proteomics approach is spatially precise and reproducible due to conservative filtering of data, important limitations of this study must be emphasized. Firstly, no fold-change cutoff was applied, and statistical significance was based only on FDR-adjusted p-values. This means that some significant proteins may show only small changes in abundance. However, in host-parasite interactions, even small changes can be biologically important, especially for parasite proteins released into host tissue around the eggs, where local effects may be strong. Therefore, these proteins were kept in the analysis, but their biological relevance should be interpreted with caution. Secondly, our approach generates a snapshot of protein presence at the moment of tissue collection and, by itself, cannot resolve the spatiotemporal dynamics of excretion/secretion or its mechanisms. Thirdly, although we eliminate egg contamination by excising intact eggs prior to tissue collection, some proteins may derive from damaged or disintegrating eggs, or possibly from adjacent parasite stages (e.g., adults) present in the vasculature. Therefore, absolute attribution of origin to an intact, living egg should be interpreted with caution and confirmed by further experiments. However, it appears that the “contamination” of our samples with somatic proteins from these sources is minimal, as ECE1, for example, is one of the most highly expressed egg peptidases in both organs (Peterková et al., 2024), yet it was not detected in any of our periovular tissue samples. This, incidentally, strongly suggests an endogenous role for this enzyme, contrary to previous beliefs based on transcriptomic studies (Peterkova et al., 2023; Konečný and Peterková, 2024; Peterková et al., 2024), and further underscores the informative value of our approach. Fourthly, to obtain an overall picture of parasite-derived proteins in both organs and phases, we did not attempt to classify eggs and their corresponding periovular tissues prior to sampling. Randomly sampled tissues were included in the analysis if they contained a visually intact egg around which a granuloma had formed. This experimental design, therefore, does not allow us to distinguish which proteins originate from which developmental stage or age of individual eggs. Finally, this study was primarily qualitative in its primary comparisons (presence/absence across infection phases in both organs), and while we performed quantitative analyses where appropriate, the current experimental design does not support inter-organ quantitative comparisons. However, these limitations do not undermine the value of our findings in detecting which parasite proteins are actually present in selected contexts.

## 5 Conclusion

Our tissue-centric, *in situ* proteomic analysis provides a fundamentally new perspective on the biology of schistosome eggs, revealing with spatial precision the repertoire of parasite-derived proteins that host tissues encounter during infection. By defining a conserved core excretome/secretome alongside organ- and phase-specific dynamics, our findings clarify long-standing discrepancies between *in vitro* ESP studies and physiological reality, highlighting that many typically intracellular proteins, as well as those lacking classical secretion signals, are indeed present at the host–parasite interface. Moreover, the distinct profiles of egg excretions/secretions in the liver and intestine challenge the traditional interpretation of “acute” and “chronic” phases in these organs. This highlights the importance of considering egg age, tissue context, and transit dynamics when interpreting egg-driven pathology and immunomodulation. Finally, our data provide a rigorously filtered, physiologically grounded resource that not only refines our understanding of schistosome egg biology but also establishes a framework for identifying the true molecular effectors shaping granuloma formation, immune polarization, and transmission-relevant processes *in vivo*.

## Data Availability

The datasets presented in this study can be found in online repositories. The names of the repository/repositories and accession number(s) can be found in the article/**Supplementary Material**.

## Supplementary material

Supplementary Figure 1 Optimization of the sample volume for the proteomic analysis. Number of schistosomal proteins identified in microdissected mouse liver tissue samples of 10, 25, and 50 × 10^6^ µm^3^ across two biological replicates. Bold values indicate the mean number of schistosomal proteins detected at each volume.

Supplementary Data Sheet 1 Numbers of schistosomal proteins. Identified schistosomal proteins in liver and small intestine tissues from individual mouse replicates across acute and chronic infection phases, including only proteins detected in at least three biological replicates (acute: 1A–5A; chronic: 1C–5C).

Supplementary Data Sheet 2 Principal component analysis. Schistosomal proteins identified in liver and small intestine tissues from individual mouse replicates during acute and chronic infection phases (acute: 1A–5A; chronic: 1C–5C).

Supplementary Data Sheet 3 Venn diagram. Table showing the overlap of identified schistosomal proteins across four groups (acute intestine, chronic intestine, acute liver, and chronic liver).

Supplementary Data Sheet 4 Small intestine. Schistosomal proteins identified in the mouse small intestine during the acute and chronic infection phases. Including only proteins detected in at least three biological replicates (acute: A; chronic: C). * and yellow highlighting indicate ambiguous proteins; red indicates statistically significant values after FDR correction.

Supplementary Data Sheet 5 Liver. Schistosomal proteins identified in the mouse liver during the acute and chronic infection phases. Including only proteins detected in at least three biological replicates (acute: A; chronic: C). * and yellow highlighting indicate ambiguous proteins; bold red indicates statistically significant values after FDR correction.

Supplementary Data Sheet 6 Selected schistosomal proteins identified in small intestine tissue. Bold red indicates statistically significant values after FDR correction.

Supplementary Data Sheet 7 Selected schistosomal proteins identified in liver tissue. Bold red indicates statistically significant values after FDR correction.

## References

[B1] Abou-El-Naga I. F. (2022). Emerging roles for extracellular vesicles in schistosoma infection. Acta Trop. 232, 106467. doi: 10.1016/J.ACTATROPICA.2022.106467. PMID: 35427535

[B2] Acharya S. Da’dara A. A. Skelly P. J. (2021). Schistosome immunomodulators. PloS Pathog. 17, e1010064. doi: 10.1371/journal.ppat.1010064. PMID: 34969052 PMC8718004

[B3] Amaral K. B. Silva T. P. Dias F. F. Malta K. K. Rosa F. M. Costa-Neto S. F. . (2017). Histological assessment of granulomas in natural and experimental schistosoma mansoni infections using whole slide imaging. PloS One 12, e0184696. doi: 10.1371/JOURNAL.PONE.0184696. PMID: 28902908 PMC5597217

[B4] Ancarola M. E. Maldonado L. L. García L. C. A. Franchini G. R. Mourglia-Ettlin G. Kamenetzky L. . (2023). A comparative analysis of the protein cargo of extracellular vesicles from helminth parasites. Life. 13, 2286. doi: 10.3390/life13122286 38137887 PMC10744797

[B5] Ashton P. D. Harrop R. Shah B. Wilson R. A. (2001). The schistosome egg: development and secretions. Parasitology 122, 329–338. doi: 10.1017/S0031182001007351. PMID: 11289069

[B6] Attenborough T. Rawlinson K. A. Diaz Soria C. L. Ambridge K. Sankaranarayanan G. Graham J. . (2024). A single-cell atlas of the miracidium larva of schistosoma mansoni reveals cell types, developmental pathways, and tissue architecture. Elife 13, RP95628. doi: 10.7554/ELIFE.95628. PMID: 39190022 PMC11349301

[B7] Bland N. D. Pinney J. W. Thomas J. E. Turner A. J. Isaac R. E. (2008). Bioinformatic analysis of the neprilysin (M13) family of peptidases reveals complex evolutionary and functional relationships. BMC Evol. Biol. 8, 16. doi: 10.1186/1471-2148-8-16. PMID: 18215274 PMC2259306

[B8] Bruderer R. Bernhardt O. M. Gandhi T. Miladinović S. M. Cheng L. Y. Messner S. (2015). Extending the limits of quantitative proteome profiling with data-independent acquisition and application to acetaminophen-treated three-dimensional liver microtissues. Mol Cell Proteomics. 14 (5), 1400–10. doi: 10.1074/mcp.M114.044305 25724911 PMC4424408

[B9] Brunet L. R. Finkelman F. D. Cheever A. W. Kopf M. A. Pearce E. J. (1997). IL-4 protects against TNF-alpha-mediated cachexia and death during acute schistosomiasis. J. Immunol. 159, 777–785. doi: 10.4049/JIMMUNOL.159.2.777 9218595

[B10] Carson J. P. Robinson M. W. Hsieh M. H. Cody J. Le L. You H. . (2020). A comparative proteomics analysis of the egg secretions of three major schistosome species. Mol. Biochem. Parasitol. 240, 111322. doi: 10.1016/J.MOLBIOPARA.2020.111322. PMID: 32961206 PMC8059868

[B11] Cass C. L. Johnson J. R. Califf L. L. Xu T. Hernandez H. J. Stadecker M. J. . (2007). Proteomic analysis of schistosoma mansoni egg secretions. Mol. Biochem. Parasitol. 155, 84–93. doi: 10.1016/j.molbiopara.2007.06.002. PMID: 17644200 PMC2077830

[B12] Chen F. Liu Z. Wu W. Rozo C. Bowdridge S. Millman A. . (2012). An essential role for th2-type responses in limiting acute tissue damage during experimental helminth infection. Nat. Med. 18, 260–266. doi: 10.1038/nm.2628. PMID: 22245779 PMC3274634

[B13] Chuah C. Jones M. K. Burke M. L. Owen H. C. Anthony B. J. McManus D. P. . (2013). Spatial and temporal transcriptomics of schistosoma japonicum-induced hepatic granuloma formation reveals novel roles for neutrophils. J. Leukoc. Biol. 94, 353–365. doi: 10.1189/JLB.1212653. PMID: 23709687

[B14] Coghlan A. Tyagi R. Cotton J. A. Holroyd N. Rosa B. A. Tsai I. J. . (2019). Comparative genomics of the major parasitic worms. Nat. Genet. 51, 163–174. doi: 10.1038/s41588-018-0262-1. PMID: 30397333 PMC6349046

[B15] Costain A. H. MacDonald A. S. Smits H. H. (2018). Schistosome egg migration: mechanisms, pathogenesis and host immune responses. Front. Immunol. 9, 424814. doi: 10.3389/fimmu.2018.03042 PMC6306409 30619372

[B16] Costain A. H. Phythian-Adams A. T. Colombo S. A. P. P. Marley A. K. Owusu C. Cook P. C. . (2022). Dynamics of host immune response development during schistosoma mansoni infection. Front. Immunol. 13, 906338. doi: 10.3389/fimmu.2022.906338. PMID: 35958580 PMC9362740

[B17] DeMarco R. Mathieson W. Manuel S. J. Dillon G. P. Curwen R. S. Ashton P. D. . (2010). Protein variation in blood-dwelling schistosome worms generated by differential splicing of micro-exon gene transcripts. Genome Res. 20, 1112–1121. doi: 10.1101/GR.100099.109. PMID: 20606017 PMC2909574

[B18] Dvořák J. Fajtová P. Ulrychová L. Leontovyč A. Rojo-Arreola L. Suzuki B. M. . (2016). Excretion/secretion products from schistosoma mansoni adults, eggs and schistosomula have unique peptidase specificity profiles. Biochimie 122, 99–109. doi: 10.1016/J.BIOCHI.2015.09.025. PMID: 26409899 PMC4747843

[B19] Gause W. C. Wynn T. A. Allen J. E. (2013). Type 2 immunity and wound healing: evolutionary refinement of adaptive immunity by helminths. Nat. Rev. Immunol. 13, 607–614. doi: 10.1038/nri3476. PMID: 23827958 PMC3789590

[B20] Gobert G. N. McManus D. P. Nawaratna S. Moertel L. Mulvenna J. Jones M. K. (2009). Tissue specific profiling of females of schistosoma japonicum by integrated laser microdissection microscopy and microarray analysis. PloS Negl.Trop. Dis. 3, e469. doi: 10.1371/JOURNAL.PNTD.0000469. PMID: 19564906 PMC2696939

[B21] Gómez-Arreaza A. Acosta H. Quiñones W. Concepción J. L. Michels P. A. M. Avilán L. (2014). Extracellular functions of glycolytic enzymes of parasites: unpredicted use of ancient proteins. Mol. Biochem. Parasitol. 193, 75–81. doi: 10.1016/J.MOLBIOPARA.2014.02.005. PMID: 24602601

[B22] Hagen J. Young N. D. Every A. L. Pagel C. N. Schnoeller C. Scheerlinck J. P. Y. . (2014). Omega-1 knockdown in schistosoma mansoni eggs by lentivirus transduction reduces granuloma size *in vivo*. Nat. Commun. 5, 5375. doi: 10.1038/ncomms6375. PMID: 25400038 PMC4243216

[B23] Harnett W. (2014). Secretory products of helminth parasites as immunomodulators. Mol. Biochem. Parasitol. 195, 130–136. doi: 10.1016/J.MOLBIOPARA.2014.03.007. PMID: 24704440

[B24] Herbert D. R. Hölscher C. Mohrs M. Arendse B. Schwegmann A. Radwanska M. . (2004). Alternative macrophage activation is essential for survival during schistosomiasis and downmodulates t helper 1 responses and immunopathology. Immunity 20, 623–635. doi: 10.1016/S1074-7613(04)00107-4. PMID: 15142530

[B25] Howe K. L. Bolt B. J. Shafie M. Kersey P. Berriman M. (2017). Wormbase parasite - a comprehensive resource for helminth genomics. Mol. Biochem. Parasitol. 215, 2–10. doi: 10.1016/J.MOLBIOPARA.2016.11.005. PMID: 27899279 PMC5486357

[B26] Huston C. D. Houpt E. R. Mann B. J. Hahn C. S. Petri W. A. (2000). Caspase 3-dependent killing of host cells by the parasite entamoeba histolytica. Cell. Microbiol. 2, 617–625. doi: 10.1046/J.1462-5822.2000.00085.X. PMID: 11207613

[B27] Ittiprasert W. Mann V. H. Karinshak S. E. Coghlan A. Rinaldi G. Sankaranarayanan G. . (2019). Programmed genome editing of the omega-1 ribonuclease of the blood fluke, schistosoma mansoni. Elife 8, e41337. doi: 10.7554/elife.41337. PMID: 30644357 PMC6355194

[B28] Jeffery C. J. Jeffery C. J. (2019). Intracellular/surface moonlighting proteins that aid in the attachment of gut microbiota to the host. AIMS Microbiol. 5, 77–86. doi: 10.3934/MICROBIOL.2019.1.77. PMID: 31384704 PMC6646928

[B29] Knuhr K. Langhans K. Nyenhuis S. Viertmann K. Kildemoes A. M. O. Doenhoff M. J. . (2018). Schistosoma mansoni egg-released IPSE/alpha-1 dampens inflammatory cytokine responses via basophil interleukin (IL)-4 and IL-13. Front. Immunol. 9, 2293. doi: 10.3389/fimmu.2018.02293. PMID: 30364177 PMC6191518

[B30] Konečný L. Jedličková L. Ibnahaten Z. Roberts A. Crosnier C. Dvořák J. (2025). Eggs-posed: revision of schistosoma mansoni venom allergen-like proteins unveils new genes and offers new insights into egg-host interactions. BMC Genomics 26, 189. doi: 10.1186/S12864-025-11369-4. PMID: 39994520 PMC11854430

[B31] Konečný L. Peterková K. (2024). Unveiling the peptidases of parasites from the office chair – the endothelin-converting enzyme case study. Adv. Parasitol. 126, 1–52. doi: 10.1016/BS.APAR.2024.05.003. PMID: 39448189

[B32] Leontovyč A. Ulrychová L. Horn M. Dvořák J. (2020). Collection of excretory/secretory products from individual developmental stages of the blood fluke schistosoma mansoni. Methods Mol. Biol. 2151, 55–63. doi: 10.1007/978-1-0716-0635-3_5. PMID: 32451995

[B33] Lu Z. Sankaranarayanan G. Rawlinson K. A. Offord V. Brindley P. J. Berriman M. . (2021). The transcriptome of schistosoma mansoni developing eggs reveals key mediators in pathogenesis and life cycle propagation. Front.Trop. Dis. 2, 713123. doi: 10.3389/fitd.2021.713123. PMID: 36389622 PMC7613829

[B34] Macháček T. Leontovyč R. Šmídová B. Majer M. Vondráček O. Vojtěchová I. . (2022). Mechanisms of the host immune response and helminth-induced pathology during trichobilharzia regenti (schistosomatidae) neuroinvasion in mice. PloS Pathog. 18, e1010302. doi: 10.1371/journal.ppat.1010302. PMID: 35120185 PMC8849443

[B35] Mathieson W. Wilson R. A. (2010). A comparative proteomic study of the undeveloped and developed schistosoma mansoni egg and its contents: the miracidium, hatch fluid and secretions. Int. J. Parasitol. 40, 617–628. doi: 10.1016/J.IJPARA.2009.10.014. PMID: 19917288

[B36] Metsalu T. Vilo J. (2015). Clustvis: a web tool for visualizing clustering of multivariate data using principal component analysis and heatmap. Nucleic Acids Res. 43, W566–W570. doi: 10.1093/NAR/GKV468. PMID: 25969447 PMC4489295

[B37] Montasser A. Dakrory A. E. Ibrahim M. I. M. Zayyat E. E. Tallima H. Ridi R. E. (2024). Differential murine responses to schistosoma mansoni eggs in the liver and small intestine lead to downmodulation of hepatic but not intestinal periovular granulomas. Infect. Immun. 92, e00362-24. doi: 10.1128/IAI.00362-24. PMID: 39560403 PMC11629614

[B38] Nawaratna S. S. K. Gobert G. N. Willis C. Chuah C. McManus D. P. Jones M. K. (2014). Transcriptional profiling of the oesophageal gland region of male worms of schistosoma mansoni. Mol. Biochem. Parasitol. 196, 82–89. doi: 10.1016/J.MOLBIOPARA.2014.08.002. PMID: 25149559

[B39] Nawaratna S. S. K. McManus D. P. Moertel L. Gobert G. N. Jones M. K. (2011). Gene atlasing of digestive and reproductive tissues in schistosoma mansoni. PloS Negl.Trop. Dis. 5, e1043. doi: 10.1371/JOURNAL.PNTD.0001043. PMID: 21541360 PMC3082511

[B40] Olivier M. Atayde V. D. Isnard A. Hassani K. Shio M. T. (2012). Leishmania virulence factors: focus on the metalloprotease GP63. Microbes Infect. 14, 1377–1389. doi: 10.1016/J.MICINF.2012.05.014. PMID: 22683718

[B41] Peterková K. Konečný L. Macháček T. Jedličková L. Winkelmann F. Sombetzki M. . (2024). Winners vs. losers: schistosoma mansoni intestinal and liver eggs exhibit striking differences in gene expression and immunogenicity. PloS Pathog. 20, e1012268. doi: 10.1371/journal.ppat.1012268. PMID: 38814989 PMC11166329

[B42] Peterkova K. Vorel J. Ilgova J. Ostasov P. Fajtova P. Konecny L. . (2023). Proteases and their inhibitors involved in schistosoma mansoni egg-host interaction revealed by comparative transcriptomics with fasciola hepatica eggs. Int. J. Parasitol. 53, 253–263. doi: 10.1016/J.IJPARA.2022.12.007. PMID: 36754342

[B43] Pino-Heiss S. Brown M. McKerrow J. H. (1985). Schistosoma mansoni: degradation of host extracellular matrix by eggs and miracidia. Exp. Parasitol. 59, 217–221. doi: 10.1016/0014-4894(85)90075-X. PMID: 3882446

[B44] Ranasinghe S. McManus D. P. (2013). Structure and function of invertebrate kunitz serine protease inhibitors. Dev. Comp. Immunol. 39, 219–227. doi: 10.1016/J.DCI.2012.10.005. PMID: 23186642

[B45] Rappsilber J. Mann M. Ishihama Y. (2007). Protocol for micro-purification, enrichment, pre-fractionation and storage of peptides for proteomics using stagetips. Nat. Protoc. 2, 1896–1906. doi: 10.1038/nprot.2007.261. PMID: 17703201

[B46] Schwartz C. Fallon P. G. (2018). Schistosoma “eggs-iting” the host: granuloma formation and egg excretion. Front. Immunol. 9, 416646. doi: 10.3389/fimmu.2018.02492 PMC6232930 30459767

[B47] Shigetomi H. Onogi A. Kajiwara H. Yoshida S. Furukawa N. Haruta S. . (2010). Anti-inflammatory actions of serine protease inhibitors containing the kunitz domain. Inflammation Res. 59, 679–687. doi: 10.1007/S00011-010-0205-5. PMID: 20454830

[B48] Silva L. M. Fernandes A. L. M. Barbosa A. Oliveira I. R. Andrade Z. A. (2000). Significance of schistosomal granuloma modulation. Mem. Inst. Oswaldo Cruz 95, 353–361. doi: 10.1590/S0074-02762000000300010. PMID: 10800193

[B49] Turner J. D. Narang P. Coles M. C. Mountford A. P. (2012). Blood flukes exploit peyer’s patch lymphoid tissue to facilitate transmission from the mammalian host. PloS Pathog. 8, e1003063. doi: 10.1371/JOURNAL.PPAT.1003063. PMID: 23308064 PMC3534376

[B50] Tyanova S. Temu T. Sinitcyn P. Carlson A. Hein M. Y. Geiger T. . (2016). The perseus computational platform for comprehensive analysis of (prote)omics data. Nat. Methods 13, 731–740. doi: 10.1038/nmeth.3901. PMID: 27348712

[B51] Vondráček O. Mikeš L. Talacko P. Leontovyč R. Bulantová J. Horák P. (2022). Differential proteomic analysis of laser-microdissected penetration glands of avian schistosome cercariae with a focus on proteins involved in host invasion. Int. J. Parasitol. 52, 343–358. doi: 10.1016/j.ijpara.2021.12.003. PMID: 35218763

[B52] Weinstock J. V. Boros D. L. (1981). Heterogeneity of the granulomatous response in the liver, colon, ileum, and ileal Peyer’s patches to schistosome eggs in murine schistosomiasis mansoni. J. Immunol. 127, 1906–1909. doi: 10.4049/JIMMUNOL.127.5.1906 6975303

[B53] Weinstock J. V. Boros D. L. (1983). Organ-dependent differences in composition and function observed in hepatic and intestinal granulomas isolated from mice with schistosomiasis mansoni. J. Immunol. 130, 418–422. doi: 10.4049/JIMMUNOL.130.1.418 6600190

[B54] Wilbers R. H. P. Schneiter R. Holterman M. H. M. Drurey C. Smant G. Asojo O. A. . (2018). Secreted venom allergen-like proteins of helminths: Conserved modulators of host responses in animals and plants. PloS Pathog. 14, e1007300. doi: 10.1371/JOURNAL.PPAT.1007300. PMID: 30335852 PMC6193718

[B55] Willett S. Olson S. A. Horejsi R. V. Nelson C. N. Wheeler N. J. (2025). Tissue-specific distribution of eggs in the definitive host drives transcriptomic and behavioral differences in Schistosoma mansoni miracidia. bioRxiv, 2025.08.15.670345. doi: 10.1101/2025.08.15.670345. PMID: 40894783 PMC12393456

[B56] World Health Organisation (2023). Schistosomiasis. Available online at: https://www.who.int/news-room/fact-sheets/detail/schistosomiasis (Accessed February 09, 2026).

[B57] Yoshino T. P. Brown M. Wu X. J. Jackson C. J. Ocadiz-Ruiz R. Chalmers I. W. . (2014). Excreted/secreted Schistosoma mansoni venom allergen-like 9 (SmVAL9) modulates host extracellular matrix remodelling gene expression. Int. J. Parasitol. 44, 551–563. doi: 10.1016/J.IJPARA.2014.04.002. PMID: 24859313 PMC4079936

