# Supplementary material for: True Schistosoma mansoni eggs-cretome revealed by laser microdissection of infected mouse liver and intestine
Source: Front Cell Infect Microbiol. 2026 May 15;16:1807773. doi: 10.3389/fcimb.2026.1807773 (PMC13219023; doi:10.3389/fcimb.2026.1807773)
Supplement: Supplementary Figure 1 — Optimization of the sample volume for the proteomic analysis. Number of schistosomal proteins identified in microdissected mouse liver tissue samples of 10, 25, and 50 × 106 µm3 across two biological replicates. Bold values indicate the mean number of schistosomal proteins detected at each volume. [file Supplementaryfile1.docx]

Supplementary Material

**
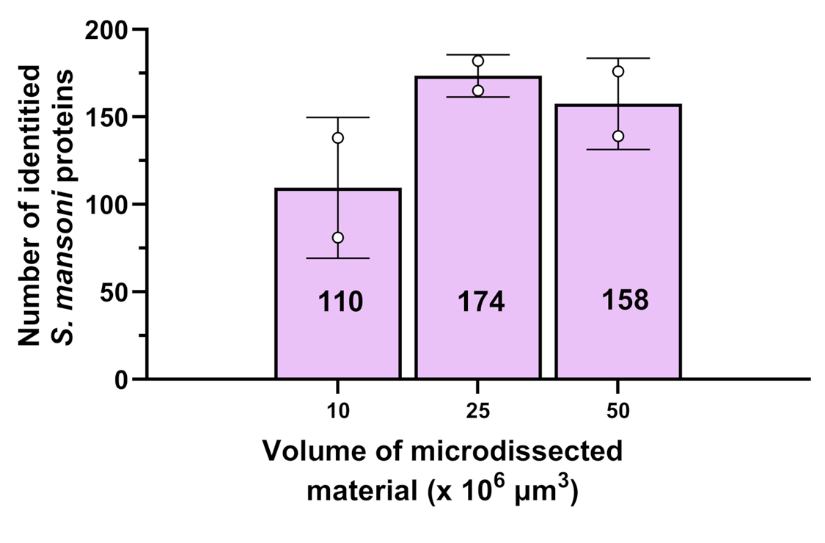
**

**Supplementary Figure 1.** Optimization of the sample volume for the proteomic analysis. Number of schistosomal proteins identified in microdissected mouse liver tissue samples of 10, 25, and 50 × 10^6^ µm^3^ across two biological replicates. Bold values indicate the mean number of schistosomal proteins detected at each volume.
